# Why did the use of antimony-bearing alloys in Bronze Age Anatolia fall dormant after the Early Bronze Age?: A Case from Resuloğlu (Çorum, Turkey)

**DOI:** 10.1371/journal.pone.0234563

**Published:** 2020-07-16

**Authors:** Gonca Dardeniz

**Affiliations:** Department of Protohistory and Near Eastern Archaeology, Faculty of Letters, Istanbul University, Beyazıt, Istanbul,Turkey; University at Buffalo - The State University of New York, UNITED STATES

## Abstract

The archaeometallurgical and archaeological research carried out in Anatolia has provided numerous examples of diverse alloying practices representing different levels of societal interaction, from the extraction of ores to the trade of finished goods and high level gift exchange among elites. While discussions abound about the exploitation of mines, mining settlements, possible origins of artifacts, resources of copper, arsenic, and especially tin to improve our knowledge about Anatolian Bronze Age mining and metallurgy, uncommon alloying practices including the use of antimony, nickel, or lead have long remained in the shadows of scholarly research. With the aim of bringing attention to the diversity in alloying practices in Anatolian metallurgy, this article focuses on the use of antimony through an appraisal of archaeological and textual evidence from Bronze Age Anatolia. Archaeometric data from several Early Bronze Age sites are re-examined alongside new data emerging from Resuloğlu (Çorum, Turkey) to explain the reduction of the variety of alloy types used. Portable-XRF analysis of artifacts from Resuloğlu and mineralogical analysis of an antimony-bearing ore fragment present evidence of use of antimony at the region during the Early Bronze Age. This period is followed by disappearance of antimony in material record until the Iron Age, while textual records weakly refer to its circulation within the region. This paper considers geological, technological, and socio-economic factors to explain why the use of antimony alloys falls dormant after the Early Bronze Age. The political and economic change towards centralization over geological and technological factors is proposed as an explanation.

## Introduction

Humans have always developed ways to manipulate the environment, often in ways unique to their landscape and social processes. Ancient metallurgical practices are one of the ways humans interact with their environment, thus stimulating archaeologists, anthropologists, material scientists, and social theorists to approach diverse aspects of the history and technology of mining [1]. From the recognition of and access to ores to the social contexts of production and economic demand, there are a wide variety of questions posed in the literature to understand ancient mining communities and their practices.

In Anatolia, early mining and metallurgy typically move through the use of copper and its alloys. The sequence of native copper, smelted copper, arsenical copper, and tin bronze has been demanding the principal attention of archaeologists and archaeometallurgists [2–3]. While scholars discussed the procurement of ores, smelting practices, or trading of tin, and tried to approach the ancient Near East as a whole, local variations received slight attention. I. Morris [4] presented the importance of local variability, which has historically caused “booms”. This article focuses on antimony as a local variant in Anatolian metallurgy. Even though antimony and antimonial alloys have not triggered a boom either for Anatolian or for ancient Near Eastern metallurgy overall, I argue, with the help of a case study, how antimony might have played a role in the diversification of alloying practices in Anatolian metallurgy. Through the review and re-examination of archaeological evidence, I demonstrate the significance of tracking and understanding diverse and local patterns of resource use in ancient societies, and propose a hypothesis explaining why the use of antimonial alloys becomes dormant and the diversity in alloying practices shrinks after the Early Bronze Age.

### Antimony: Material characteristics and history of use

References to the use of antimony in Anatolia are scattered throughout the literature of archaeology, philology, and geology. This study evaluates archaeological, archaeometric, textual, and to a certain extent, geological data to understand the use of antimony in Bronze Age Anatolia, specifically focusing on the Early Bronze Age (ca. 3100–2050/2000 BC). While reassessing the broader region of the Near East, I present recent archaeometric data from the Early Bronze Age site of Resuloğlu (Çorum, Turkey).

### Material characteristics

Antimony (Sb) is a lustrous grey, white metalloid with the atomic number 51 and an atomic weight of 122. It has a moderate melting point of 630°C; elemental antimony is brittle, with a hardness of three according to Mohs scale, which renders it too soft to manufacture hard objects from it.

In the periodic table antimony is located below arsenic (As), which has an atomic number of 33 and Mohs hardness 3.5. These two metalloids share some chemical characteristics. Antimony, like arsenic, gives hardness to copper in alloying. The addition of more than 3–7 wt% antimony makes alloys harder than pure copper to produce sharper tools. Alloys containing more than 7 wt% of antimony cause antimony to separate from the matrix causing a weakening in the material [2: p.171–172]. Alloys with an antimony content between 10–25 wt% are brittle but make casting alloys of tempting appearance [5–6]. Antimony provides hardness to metals like tin and lead while decreasing the degree of oxidation. Aside mechanical properties, antimony affects color of its alloys with copper. Copper–antimony alloys with higher than 6–7 wt% of antimony turn their color towards blue as antimony content increases [7].

The average grade of antimony in the earth’s crust is documented variably from 0.4 [8] to 0.65 mg/kg [9]. It has rarely been found in nature as native antimony (i.e., 100% antimony) due to its high affinity to bond to sulfur. It easily compounds with metals like copper, lead, and silver. Antimony forms a wide range of minerals from native antimony to complex oxides, hydroxides, and sulfosalts [9: p.188]. There are more than a hundred antimony minerals in nature; among these, stibnite is the most abundant [10], (**Table 1**). Commonly, stibnite (Sb_2_S_3_) is associated with arsenopyrite, an iron arsenic sulfide (FeAsS) with silvery to blackish sheen [9].

**Table 1:** Selected primary antimony minerals. Confirmed archaeological use (a) in Armenia [11]; (b) in Iran [12]; (c) in Nahal Mishmar (tennantite–tetrahedrite series Cu_12_As_4_S_13_–Cu_12_Sb_4_S_13_) [13: fig. 30].

Mineral	Formula
Stibnite (Antimonite)	Sb_2_S_3_
Boulangerite (a)	Pb_5_Sb_4_S_11_
Luzonite (b)	Cu_3_AsS_4_
Cervantite	Sb_2_O_4_
Senarmontite	Sb_2_O_3_
Valentinite	Sb_2_O_3_
Tetrahedrite (c)	Cu_12_Sb_4_S_13_
Jamesonite	Pb_4_FeSb_6_S_14_
Burnonite	PbCuSbS_3_
Gudmundite	FeSbS
Livingstonite	HgSb_4_S_7_
Kermesite	Sb_2_S_2_O
Berthierite	FeSb_2_S_4_
Allemontite	AsSb
Stibiconite	Sb_2_O_4_.H_2_O

Antimony deposits are classified into three major groups: 1) magmatic, 2) structure-related, and 3) sedimentary [9: table 21.01]. While this classification has further geological subgroupings, Anatolian antimony deposits include both sedimentary and magmatic formations besides structure-bound; polymetallic deposits are available both in western and central Anatolia [9; 14]. The most common antimony minerals in Anatolia are stibnite (Sb_2_S_3_), valentinite (Sb_2_O_3_), and senarmontite (Sb_2_O_3_). In 2002, the metallic antimony reserve of Turkey was estimated to be 106,306 million tons [15: p.94]. The variety and wealth in deposits list Turkey as the second biggest producer of antimony after China [16]. The discussion of all antimony occurrences in Turkey is beyond the subject of this research, hence some of them with relevance to Bronze Age are discussed below.

### History of use: Material and textual evidence

Even though antimony has similar chemical characteristics to arsenic, archaeometric research demonstrates that antimony has not been used as commonly as arsenic. Since objects with a high antimony content are soft, they are not appropriate for tools or weapons, thus artifacts containing antimony are generally used for ritual or small items such as jewelry. In this section, I present a broad spatial (Anatolia, Mesopotamia, the Levant, and Caucasus) and temporal (Late Chalcolithic to Iron Age) review of antimony-bearing artifacts to discern their role in ancient Anatolia better.

In Mesopotamia and Egypt, stibnite was used in conjunction with lead for eye cosmetics called kohl [17: p.80–83]. In Mesopotamia, there is evidence for the use of antimony as early as the 3^rd^ millennium BC. The archival resources from Ebla, to the south of Anatolia, which date to the second half of the 3^rd^ millennium BC mention antimony. In the tablet TM.75.G.2154, 41.20 minas, roughly equal to 19.43 kg, of antimony paste were recorded for the manufacture of a jar [18: p.189–190]. A bead found at Tell Leilan was found to be composed of 99.7% antimony. In Jerablus Tahtani (Syria) a bead containing 98.5% antimony and traces of copper, arsenic, tin, and lead was recovered at levels dating to 2500–2300 BC [19]. A similar find dated to circa 2000 BC was recovered in a funerary context from Aššur [20: p.242]. I. R. Selimkhanov [21] analyzed a pure antimony vase fragment (?) and suggested a southern Caucasus origin for antimony, however the results were accepted as inconclusive due to the fragile nature of antimony, which would impede its ability to form a vase [20: p.241].

In Norşuntepe (southeastern Anatolia), slags found in the Uruk settlement (ca. 3500 BC) were analyzed. It was discovered that a crystalline type of ore including antimony, as well as arsenic, could have been used as a raw material to alloy copper [22: p.17], (**S1 File)**, (**Tables 2 and 3**). This type of antimony, as well as arsenic, lead, and zinc are known and could be sourced from the Azerbaijan mineral zone, which is the continuation of the northeastern Anatolian zone [23: p.66]. In the Late Chalcolithic strata of the settlements no evidence of antimony or arsenic has been reported; this dearth is associated with a change in the metallurgy at the site [22: p.17].

**Table 2:** Chronology used throughout the text.

Period	Chronology
Late Chalcolithic	3590–3470 BC
Early Bronze Age I	3100–2700 BC
Early Bronze Age II	2700–2450 BC
Early Bronze Age III	2450/2400–2050 BC
Middle Bronze Age	2050–1719/1685 BC
(Old Assyrian Trading Colony)	
Late Bronze Age	1550/1525–1190 BC
Iron Age	1190–5^th^ century BC
Early Iron Age	12–10^th^ century BC
Middle Iron Age	9^th^ century BC–ca. 650 BC

**Table 3:** List of antimony-bearing finds (ores, slag fragments and artifacts) from the Near East and Caucasus (with additions after [32]: p.199, table 1).

	Site	Region	Period	Finds	Reference
1	Norşuntepe	Anatolia	Uruk	Slag, ore fragments	[22]
2	Arslantepe	Anatolia	Uruk, Late Uruk	Slag, ore fragments	[33]
3	Nahal Mishmar	Israel	Chalcolithic	Crowns, standards, mace heads	[13]; [36]; [37]
4	Nahal Zeelim	Israel	Chalcolithic	Mace heads	[6]; [32: p.199, table 1]
5	Negev	Israel	Chalcolithic	Mace head	[38]
6	Nahal Qanah	Israel	Chalcolithic	Lump of metal	[39]
7	Bir es-Safadi	Israel	Chalcolithic	Block of metal	[6]; [40]
8	Palmahim	Israel	Chalcolithic	Mace head	[32: p.199, table 1]
9	Arslantepe	Anatolia	EBA I	Beaker	[33]
10	Tarsus-Gözlükule	Anatolia	EBA II	Two stamp seals	[32: p.200, table 3]
11	Polatlı	Anatolia	EBA	Piece of a vessel	[42]
12	Ahlatlıbel	Anatolia	EBA	Bead	[42]
13	Troas, NW Anatolia (assemblage of metals)	Anatolia	EBA II/III	Vase	[42: p.143, 172]; [45]
14	Troy	Anatolia	EBA II/III	Vase, flat axe	[45: p.578–579]
15	Soloi	Anatolia	EBA III	Stamp seal, dagger	[59]; [61]
16	Tepecik	Anatolia	EBA	Pin	[98: table 3]
17	Resuloğlu	Anatolia	EBA III	Beads, ornaments	[51]; [94]
18	Alaca Höyük	Anatolia	EBA III	Figurine	[56]
19	Kültepe	Anatolia	EBA	Pin	[57: p.199, table 1]
20	Tell Abu Hgaira	Syria	EBA	Bead	[20]
21	Tell Leilan	Syria	EBA	Beads	[20]
22	Jerablus Tahtani	Syria	EBA	Bead	[19]
23	Tello	Iraq	EBA	Vase (?) fragment	[21]
24	Velikent/Daguestan	Caucasus	EBA	Beads, buttons, and pendants	[21]
25	Mamai-Koutan/Daguestan	Caucasus	EBA	Beads, buttons, and pendants	[21]
26	Abkhazia	Caucasus	MBA	Pendants	[71]
27	Brili	Caucasus	MBA; LBA	Buttons, Axe, and pendants	[32: p.199, table 1]; [35]
28	Kwasatali	Caucasus	MBA	Buttons and pendants	[32: p.199, table 1]
29	Zagwli	Caucasus	MBA	Pendants	[32: p.199, table 1]
30	Bornighele	Caucasus	MBA	Axes, mace heads, beads	[32: p.199, table 1]
31	Aššur	Iraq	MBA	Buttons	[20]
32	Lchashen and Artik	Armenia	MBA–LBA	Various	[11]
33	Balıklı (Şavşat, Artvin)	Anatolia (Black Sea)	LBA	Axe	[72]; [42: p.127]
34	Kayakent	Caucasus	LBA	Beads, buttons, and pendants	[71]
35	Redkin	Caucasus	LBA	Beads, buttons, and pendants	[71]
36	Treli	Caucasus	LBA	Buttons and pendants	[32: p.199, table 1]
37	Tschalipiragorebi	Caucasus	LBA	Beads	[32: p.199, table 1]; [35]
38	Meligele	Caucasus	LBA	Buttons	[71]
39	Tell el-Farah	Levant	IA	Beads (66% tin, 33% antimony)	[20: p.241]
40	Hasanlu	Iran	IA	Pendants	[78]
41	Lahun	Egypt	IA	Beads	[20]
42	Yoncatepe	Anatolia	IA	Buttons	[76]
43	Erzurum and Kars Museums collections	Anatolia	LBA–EIA	Various (daggers, axes, arrowheads, swords)	[74]
44	Metsamor	Armenia	LBA–EIA	Various	[11]
45	The British Museum collections	Caucasus	LBA–EIA	Various artifacts in the British Museum collections belonging to the Koban and Transcaucasian culture	[77]
46	Chambarak (Krasnoselsk) burials	Armenia	EIA–MIA	Buttons (pure antimony and Sb+Pb alloy)	[11]; [75]
47	Bjni tombfield	Central Armenia	EIA–MIA	Button	[11]

EBA: Early Bronze Age; MBA: Middle Bronze Age; LBA: Late Bronze Age; EIA: Early Iron Age, MIA: Middle Iron Age. The tripartite system for EBA as EBAI–II–III is followed where chronological data allows. Compositional details and types of instrumental analyses (if given) can be found in the listed references.

In the northern Anatolia, the site İkiztepe situates where Halys River meets the Black Sea. The site consists of four mounds despite the fact that İkiztepe means “twin mounds”. The Late Chalcolithic levels of İkiztepe mound II yielded one sample out of 44 with 1.3% antimony and 1.71% arsenic [24: table 1]. However, the total weight percentage of this sample far exceeds 100%, making the result debatable. The rest of the assemblage contains pure copper objects with less than 1% arsenic and trace amounts of antimony [25: p.68]. Some slag samples collected from the vicinity of the site with uncertain chronology revealed antimony-bearing prills [25: p.72, 79, table 2]. The majority of the metal artifacts from İkiztepe were recovered at the cemetery area in mound I (i.e., İkiztepe I) [26–28]. While an extensive analytical project on 360 metal objects, ores sources, and metallurgical debris has been on-going since the 1980s, preliminary reports documented the use of arsenical copper with significant levels of nickel [25; 29]. This cemetery, thus the accompanying metal artifacts, was dated to the Early Bronze Age III based on relative and radiocarbon dating [25; 26: p.144; 27; 28: p.67]. However, recent research and three radiocarbon dates from human remains pulled the chronology of the cemetery back to the Late Chalcolithic-Early Bronze Age transitional period [30: p.105; 31: p.143]. The chronological controversies and the compositional analyses await detailed publications for a clearer understanding of the site and its metallurgy.

**Fig 1:**
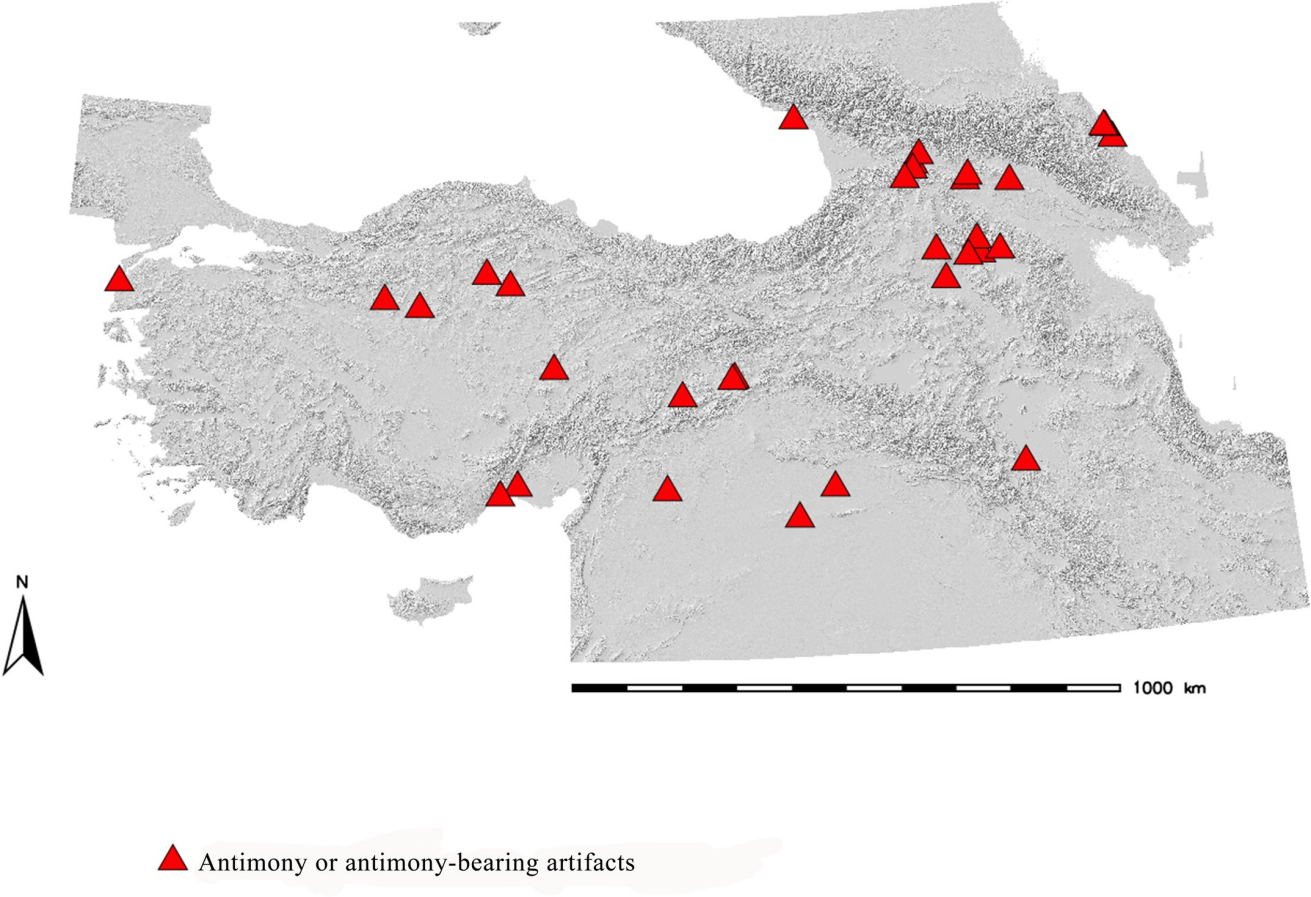
Dispersal of antimony and antimony-bearing objects from the Early Bronze to the Early Iron Ages with the sites listed in Table 2 (map: Courtesy of Bülent Arıkan). For a broader regional map covering Chalcolithic finds and resources see: [35: fig. 1]. Details of Anatolian sites are provided in S1 Fig.

In Arslantepe (Malatya, Turkey), copper–arsenic–antimony-bearing ore fragments (inventory no’s: 90/129 E6(1) 9b; 91/338 E6(3) A657/P1; E6(3) 4a; E6(3)–E5 (15) A657 rP1) were found containing as much as 7% antimony in levels VII (Late Chalcolithic, ca. 3750–3400 BC) and VIA (inventory no: 93/156 E9(3) 33a; Late Uruk, ca. 3400–3000 BC) along with copper-, arsenic-, lead-, iron-, and nickel-bearing ore examples. Two lead slag from Level VII (inventory no’s: D5 (10) 1A; C19(5) 3c27) contain 7.65% and 5.41% antimony [33: p.48, 54, tables 4, 8]. These ore fragments could be used for other purposes (i.e., pigments) rather than metal production. They only confirm existence/access to antimony-bearing polymetallic ores during the 4^th^ millennium BC. In the latter period, a beaker (excavation no: ARSL 58) from the Royal Tombs of Arslantepe (Level VI B1, Early Bronze Age I, ca. 3000–2700 BC) shows an unusual composition of copper–arsenic–antimony–iron with 2.74% antimony, 8.66% arsenic, and 4.61% iron (**Fig 1**) (**S1 Fig**). Even though, Hauptmann et al. [33: p.52] suggested some antimony-rich ore samples from level VII as possible preparatory material for such metal objects, archaeometric data did not support this argument. Nevertheless, the existence of complex copper ores with high antimony, arsenic, nickel, and lead could be related to experimentation with polymetallic ores leading to the production of alloys with diverse compositions in Arslantepe [33: p.65; 34].

Dating to the Late Chalcolithic, Nahal Mishmar (Israel) finds provide one of the most significant groups of antimony-containing artifacts, specifically ternary alloys of copper, antimony, and arsenic. These objects are mostly elaborate and symbolic, and some of these contain as much as 26% antimony [13; 36; 37]. The positive correlation of antimony with silver, bismuth, and arsenic in these objects suggests that the metal was derived from fahlerz type copper ores rather than a mixture of pure copper and antimony–arsenic rich mineral [13: fig. 3.1]. The alloy was foreign to the region, and sulfidic inclusions suggest that the complex alloys were derived from the use of minerals tennantite–tetrahedrite (Cu_12_As_4_S_13_–Cu_12_Sb_4_S_13_). These mineral series are not available in the Levant’s major copper deposits, leading Tadmor et al. [13: fig. 30] to point to southern Caucasus, the Little Caucasus in Azerbaijan, and the southern slope of the Great Caucasus in Georgia, as possible sources.

In the same region, a mace head dated to the Chalcolithic period (4500–3500 BC) in Negev (Shiqmim, Israel) produced with a lost wax technique was documented as containing varying amounts of antimony. The archaeometric analyses conducted on different metallurgical phases of the mace head demonstrated that the α phase was made of copper alloyed with up to 2% antimony and approximately 1.5% arsenic. The interdendritic phase contains 8% antimony, 5% arsenic, and 2.4% silver [38: p.68, table 2). The co-occurrence of antimony and arsenic together with traces of silver, nickel, lead, and bismuth signifies smelting of a fahlerz type ore containing tetrahedrite (Cu_3_SbS_3_) and chalcocite (Cu_2_S) to produce the mace head. Lumps of metals containing 5% antimony, 2% arsenic, and 1% lead at Nahal Qanah cave tombs, and a block of metal from Bir es-Safadi with 2.5% antimony, 0.8% arsenic, and 1.5% lead add further evidence for the use of antimony- and arsenic-rich metals at the Levant during the Chalcolithic period [6; 39; 40]. As there are no known sources of copper–arsenic–antimony at the Levant, the origin of the metals are linked to Anatolia or Azerbaijan [6; 41]. J. Golden [6: p.574] noted the disappearance of copper alloys with antimony and arsenic at the beginning of the Early Bronze Age in the Levant, which he explained with the interruption in trade networks.

**Fig 2:**
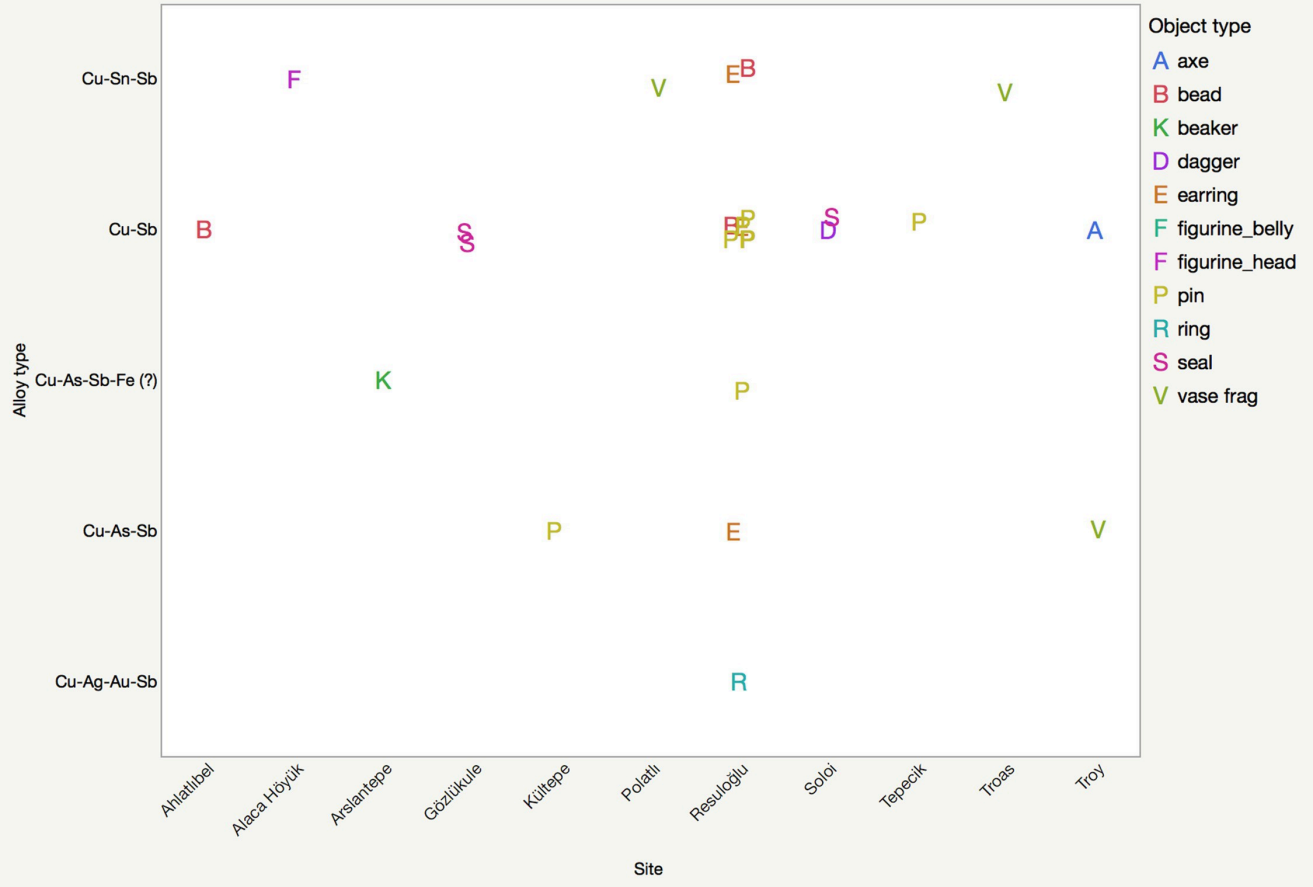
Antimony-bearing alloys and artifact types from the Early Bronze Age levels of Anatolian sites mentioned in the text. Please note that the F-labels for figurine_belly and figurine_head superimpose on the chart.

In central Anatolia, antimony-bearing artifacts have been known since the Early Bronze Age (**Figs 1, 2**), (**Tables 2 and 3**), (**S1 Fig**). Ahlatlıbel in Ankara yielded a bead including over 5% antimony, as well as lead [42: p.121, 177]. The assemblage of Ahlatlıbel includes double-spiral and hammered-headed pins, which were typologically linked to the north-central Anatolian metallurgical tradition [43: p.34]. Based on the availability of local reserves, P. De Jesus [44: p.125] suggested the metal resources at Çorum or Tokat as possible origins, if Ahlatlıbel had its own local metal industry. A spouted vase fragment recovered from the Early Bronze Age levels of Polatlı (Ankara) contains approximately 5.2% antimony [42: p.141, 171]. The 5% tin content of this fragment is also notable. Another spouted vase fragment from Troas (Biga Peninsula, northwest Anatolia) dating to the Early Bronze II/III was found to include 1.35% antimony with approximately 9.1% tin [42: p.143, 172]. Neutron activation analysis of two copper artifacts (HDM 253, HDM 267) from Hisarlık, Troy (northwest Anatolia) in Staatliche Museen, Berlin collections showed antimony contents of 1.2% and 1.7%, respectively [45: p.575, 578–579, tables 3, 4].

During the latter half of the 3^rd^ millennium BC (i.e., EBAIII), copper–tin alloy along with arsenical copper and unalloyed copper dominate the Anatolian metal collections. Alaca Höyük (Çorum) is one of the unique sites for Anatolian metallurgy with the extravagant amount and variety of its grave goods made of copper and its alloys, gold, silver, gold-silver alloys, and even iron. These metal artifacts were recovered in 13 so-called ‘royal’ graves [46; 47]. The stratigraphy concerning the relative chronology of the graves within the Early Bronze Age, however, is a much-debated issue [48: p.81; 49]. A group of wood, textile, and grain samples collected during the 1930s–1940s excavations were ^14^C-dated to address this problematic. While the preservation conditions, thus the suitability for radiocarbon dating, of the samples are unclear, the results propose ca. 2750–2500 BC for the earliest levels of the graves [50: p. 93–94, abb.5]. A burnt tree sample from a layer above the graves was dated to ca. 2450–2300 BC [50: p.94, fn.2], though the stratigraphy of the sample is ambiguous. Contemporaneous local and regional material, especially local ceramics of the Halys Basin, strongly suggests the second half of the 3rd millennium BC for the graves. The complex chronological problems of the region are beyond the coverage of this article. Further research is mandatory to fine-tune the stratigraphy of Alaca Höyük as well as other sites (e.g., Horoztepe, Mahmatlar) in central Anatolia, where the chronology of the settlements from earlier excavations heavily depend on Alaca Höyük data [31; 51].

A figurine (7027) from Alaca Höyük presents significant results regarding the use of antimony. This figurine was found in grave H (Level III, phase 5), one of the richest tombs of Alaca Höyük belonging to a female. While no radiocarbon dating from grave H is available, samples from the same level (i.e., Level III, phase 5) strongly support dating of the grave to the second half of the 3^rd^ millennium BC [50]. Furthermore, direct parallels of black burnished ceramics from this grave were uncovered in the cemetery and settlement areas at Resuloğlu and dated to circa 2500/2400–2100/2050 BC [52–55]. The X–ray fluorescence analysis (pXRF) of the figurine reveals 5.5–6.9% antimony in its belly and head, respectively. The figurine contained varying amounts of tin (8.8–10.4%), in addition to 1.5% arsenic. At the Early Bronze Age graves of Alaca Höyük, a total of seven figurines have been discovered. According to the pXRF results, figurine 7027 is the only one containing antimony that has been identified thus far. Recognizing the problem of segregation of antimony and tin on the surface, this figurine is not only compositionally different. The figurine’s smaller size and body features significantly differ from the other figurines. Considering both typological features and compositional analysis, Alaca Höyük figurine 7027 was proposed to be an import, without identifying any particular region as its possible source [56: p.45].

Karum–Kanešh, located in Kültepe (Kayseri) in central Anatolia, is well known as the center of trade between Aššur and Anatolia during the first quarter of the 2^nd^ millennium BC. While less is known about the Early Bronze Age levels of the site, 70 objects, largely consisting of small tools, pins, and sheet metal, dating to the Early Bronze III period were collected at the monumental architecture on the mound from the levels 13 through 11b. The pXRF analysis of these metals identified mostly copper, arsenical copper, and bronze (copper–tin) with an average of 7% tin, as well as the less common alloys copper–arsenic–tin and copper–arsenic–lead [57: p.196–204]. Among the artifacts, a pin shaft (Kt 22–238 B) found at the mound level 11b was documented as containing 1.34% antimony with 1.13% arsenic [57: p.199, table 1].

Other indisputable evidence for the use of antimony in Anatolia was documented at Tarsus–Gözlükule in Cilicia (southern Anatolia). Metallurgical examinations of two stamp seals (inventory no’s: T47–87; T47–1) recovered from the Early Bronze II levels at Tarsus–Gözlükule were found to be produced with a cementation technique leading to an antimony content of around 10–14% [58]. Özbal et al. [32] proposed the use of local antimony deposits from Niğde Massif in Bereketli Maden or Örendere, located approximately 140 km north of Tarsus, for the production of these seals. X–ray diffraction analysis of ore samples collected from these regions confirmed the existence of stibnite containing lead as an impurity. This strongly points towards the use of central Tauride resources [32: p. 204, table 5.7]. A stamp seal (Nr. 3394) similar to the examples from Tarsus–Gözlükule was documented at Soloi (approximately 60 km west of Tarsus) and contained 2% antimony [59]. The Soloi stamp seal differs from the Tarsus–Gözlükule examples in its lower level of antimony. A dagger (Nr. 3400a) from the site was also found as antimonial copper with 2.5% antimony and 97.45% copper [59: p.201]. The primary dating of the artifacts to the Middle Bronze Age [59] had changed to the Early Bronze Age III (ca. 2300/2200–2000 BC) based on regional typological studies of the weapons found in association [60; 61: p.7, 10]. Tarsus-Gözlükule and Soloi add another line of evidence to the recognition of antimonial alloys in the southern Anatolia during the Early Bronze Age.

The material evidence regarding the use of antimony in the Middle Bronze Age Anatolia remains sparse. Our knowledge about the existence and recognition of antimony or antimony-bearing ores as distinct materials comes from textual records. The earliest textual records in Anatolia date to the first quarter of the 2^nd^ millennium BC and demonstrate intensive trade between Aššur (northern Mesopotamia, Iraq) and Karum–Kanešh. Approximately 23,000 cuneiform tablets written in Old Assyrian from Karum–Kanešh document the details of this long-distance trade in which tin, textiles, silver, wool, and partly copper played a major role. Among these goods, tin and textiles were traded from Aššur for Anatolian silver and copper. It is important to note here that K. R. Veenhof [62: p.86] disagrees with the proposed export of copper from Anatolia to Aššur, asserting that textual records do not support it. He argues that the price of copper and cost of its transport would have inhibited its export to Aššur, even though Assyrians were involved in the copper trade within Anatolia. In lieu of extensive trading of copper between Anatolia and Aššur, Veenhof [62] suggests that traders exported small amounts, which could also have been important for the Anatolian antimony trade. Written documents must speak of antimony-bearing ores (e.g., stibnite) rather than metallic antimony. Thus, while referring to textual records in the following paragraphs, this distinction should be noticed.

Antimony was not among the goods imported from Aššur, though it was traded by Assyrians in Anatolia [62: p.85]. Eighteen tablets from Karum–Kanešh mention antimony (*lulûm*) with a total weight of 20 talents and 53 minas, approximately equal to 630 kg [63]. The reference to antimony is mostly given in copper trade texts leading scholars to suggest that *lulûm* was used in alloys as an alternative to tin and arsenic [63: p.801, fn.91]. Thus, this trade confirms the existence and use of antimony in the region.

The Assyrian word for antimony is *lulā’um* (*lulu*, *luliu*); it was listed in price lists as well as glass making and medical texts [64: p.243]. Several Akkadian words were proposed to translate to antimony: *guḫlu* as the raw material for kohl or antimony paste, as well as eye make-up; *egû* as antimony–paste–, kohl; *ṣadīdu* as for antimony; and *šimbizidû* as antimony paste. In the Ur III period, *su*.*gan or sù*.*gan* have been proposed as words for antimony, though the use of these words to mean arsenic or slags from bronze production has also been suggested [65: p.140].

A few Kültepe tablets also refer to antimony. A treaty (KTS 7a: 4) between Adad-şulūlī, a well-known medium rank merchant, and Buzāzu documented a price of 20 minas (approximately 10 kg) of copper per talent (approximately 30 kg) of antimony [66: p.62, here the names were documented as Adad-iqbi and Buzazu] [67: p.99, 138, fn. 428]. This suggests that copper was almost three times more expensive than antimony. While tin was seen as highly valuable as an alloying metal during this period, antimony seems not to have been in high demand despite its low price and easy accessibility.

J. G. Dercksen [67: p.106] stated that the cities in the west and northwest of Karum–Kanešh, such as Wahšušana, Purušhattum, and Turhumit (Durhumit) had been the foci of Adad-şulūlī’s trading activities. In a tablet (kt a/k 265), Adad-şulūlī was asked to send two minas of native copper and ten minas of antimony to Kanešh [67: p.107]. Tablets AKT 3, 52:2 and Kt k/k 79: 1f referred to two and a quarter talents of antimony, 41 talents of copper, as well as more than nine talents of antimony, respectively. The wealth of a dead trader recorded in Kt m/k 1: 34f includes two talents and ten pounds of antimony [62: p.86, fn. 357]. The highest amount of antimony was noted in tablet Kt h/k, which mentions a plan of importing ten talents of antimony from Hattum to sell it in Luhusaddia [68]. Unfortunately, tablets do no describe the details of the antimony trade [62: p.85]. Yet, archaeological evidence does not prove the circulation of such amounts of antimony either, which might be due to dearth of analytical research on the metal assemblages of major Old Assyrian trade centers.

It is essential to note here that the pXRF analysis of metal collections from Karum–Kanešh demonstrated use of unalloyed copper, arsenical copper, tin bronze, and ternary alloy of copper–arsenic–tin [57]. Yet, the majority of the collection stays unpublished, Lehner et al. [57] argued adoption of tin bronzes at Kültepe circa the late 3^rd^ millennium BC. Well-established economic institutions supporting predictable access to tin (along many other materials) through an exchange system must have raised tin use in the following periods [57: p.205]. This might have resulted in circulation and use of alloying materials such as arsenic and antimony in smaller amounts, since the economic system majorly relied on tin and its trade. This shift towards tin bronze is discussed below within the frame of diffusion of innovations (DOI) and lock-in effect.

While textual records refer to antimony trade in the Middle Bronze Age, no word for antimony is known from the subsequent Hittite period. A. M. Polvani [69: p.59–63] proposed the word ‘lulluri-’ to mean antimony. Even though it has affinity to the Akkadian *lulu*, ‘lulluri-’ is most often translated as iron or hematite with questionableness [70: p.118, HED L].

While use of antimony is unknown in central Anatolia during the Middle and Late Bronze Ages, a consumption boom of antimony and its alloys is visible at various sites in the Caucasus, where geological and archaeological research has located rich metallic reserves [35; 71] (**Table 3**). E. Chernykh [71: p.60] documented rich antimony deposits at Zopkhito in Gornaya, Georgia. While the majority of the objects are small ornaments, such as beads, pendants and buttons, limited numbers of axes and mace heads made of antimonial copper were also identified in the region. A Late Bronze Age antimonial copper axe was found in Balıklı in the Şavşat province of Artvin (the Black Sea region) situated in close proximity to the Caucasus. The axe contains 1.6% antimony and 2.6% arsenic. It was found in a collection of axes, which also contained an ingot dating to the Late Bronze Age [42: 127; 72]. While K. Bittel [38] dated the artifacts to the second half of the 2^nd^ millennium BC, recent research suggested an age of 1200–1000 BC for typologically similar artifacts found in the Çoruh River Valley and displayed in the Batum Museum [73].

Establishing a metallurgical link between Balıklı and the Caucasus appears tempting, however the understudied northeastern zone of the Black Sea region of Turkey lacks the necessary evidence. The relation of the southern Caucasus to eastern Anatolia is mostly proposed through typological studies. Nonetheless, recent research conducted with the aid of pXRF on 122 artifacts from Erzurum and Kars Museums hints existence of antimony-bearing alloys. Even though only ten of those objects, mostly weapons, were collected through systematic excavations, typological examinations date them to the Late Bronze–Early Iron Age transition period. The results show the highest antimony concentration on a dagger (museum no: 8–6–2003) from the Kars Museum. In the Erzurum Museum sample, the highest amount of antimony was detected at 1.07%, also on a dagger (museum no: 127–94) [74]. Based on the fact that these artifacts of the local museums have been unearthed from the region, the use of antimony appears disparate and non-systematic.

While Black Sea region of Turkey lacks the necessary systematic evidence, isotopic, mineralogical, and geochemical research at Georgian ores from Racha-Lechkumi and the Late Bronze Age antimony objects (ca. 15^th^ century BC) from the sites of Brili and Chalpiragorebi present promising results on use and provenance of antimony. Recent studies at the region indicate that local stibnite and tetrahedrite-tennantite could have been used as raw materials for metal and glass making. As the research regarding the use of antimony isotopes is very recent, more data from the region and its neighbors would be helpful to acknowledge the full picture [35].

In Armenia, antimony appears both as unalloyed and alloyed during the Late Bronze and Early Iron Ages. Copper–antimony and copper–antimony–lead alloys were documented even though the majority of metal corpus consists of copper–tin alloys [75]. The use of antimony continued in the Iron Age. The Urartian cemetery in Yoncatepe Kalesi in Van (Turkey) yielded antimony buttons made from sulphur-bearing antimony ore, such as stibnite [76: p.188, fig. 9]. Similar antimony and antimony–lead alloy buttons were documented as burial goods from Middle and Late Iron Age Armenia [11]. XRF and inductively coupled plasma–atomic emission spectroscopy analyses conducted on the Caucasian metalwork in the British Museum demonstrate the utilization of antimonial alloys, mostly containing 5–10% antimony [77: p.90, 96, table 2]. A. Pike [77] advocated for the deliberate use of antimony in Koban and Transcaucasian metalwork from the Late Bronze and Early Iron Ages. Small personal ornaments of 96–99% antimony and antimony rich alloys were also reported at Hasanlu in Iran [78; 79].

While antimony is important in Bronze Age metallurgy, it is also a significant player in glass and glazing technology. During the Late Bronze Age, human-made glass technology becomes fully developed as is apparent from the types, number, and colors of artifacts. Early glass, with its high-status connotations, contains vivid colors leading to its appreciation as semi-precious stone [80]. Colors are achieved via colorants and opacifiers; antimony was the earliest opacifier for glass [81]. Degryse et al. [82] suggested the Caucasus as a possible source of antimony for Late Bronze Age Mesopotamian and Egyptian glass, though it should be noted that their dataset does not include/extend to Anatolian reserves. Yet, recent research on antimony isotope presented promising results to provenance antimony in Georgian Late Bronze Age metal and glass objects [35].

Based on lead isotope research on polychrome glazes of Neo-Babylonian reliefs (ca. 605–562 BC), Rodler and colleagues [83] tentatively proposed the Taurus Mountains as the origin of antimony-based opacifier for yellow glazes. This argument is in concordance with Özbal et al.’s [32] findings for the use of Taurus stibnite as early as the Bronze Age. The use of ores, minerals or metal oxides, especially copper, antimony and lead, as colorants and opacifiers in glass has also led to discussions on the relationship of metal and glass technologies [84, with references therein]. Recent research on tracing antimony and provenancing glass through antimony, neodimyum, and strontium isotopes opens new venues to discover links between metal and glass technologies, as well as into the sourcing of antimony [35; 82; 85].

### Case site: Resuloğlu

Among the Early Bronze Age settlements containing antimony-bearing objects in Anatolia, Resuloğlu (Early Bronze II–III, ca. 2500/2400–2100/2050 BC) currently provides the highest number of artifacts. I argue that this is the result of an investigation of the complete metal corpus at the site. Since 2016, analytical research on Resuloğlu covers the metal corpus as a whole without utilizing selective sampling of extravagant metal objects, most of which were collected from burial contexts. Though, a similar analytical strategy is not common among many excavations conducted in Anatolia. The selective sampling produces an incomplete representation of metal objects at sites from Anatolia (and beyond), and favors those with tin and arsenic concentrations in alloys, which are frequently found in graves.

**Fig 3:**
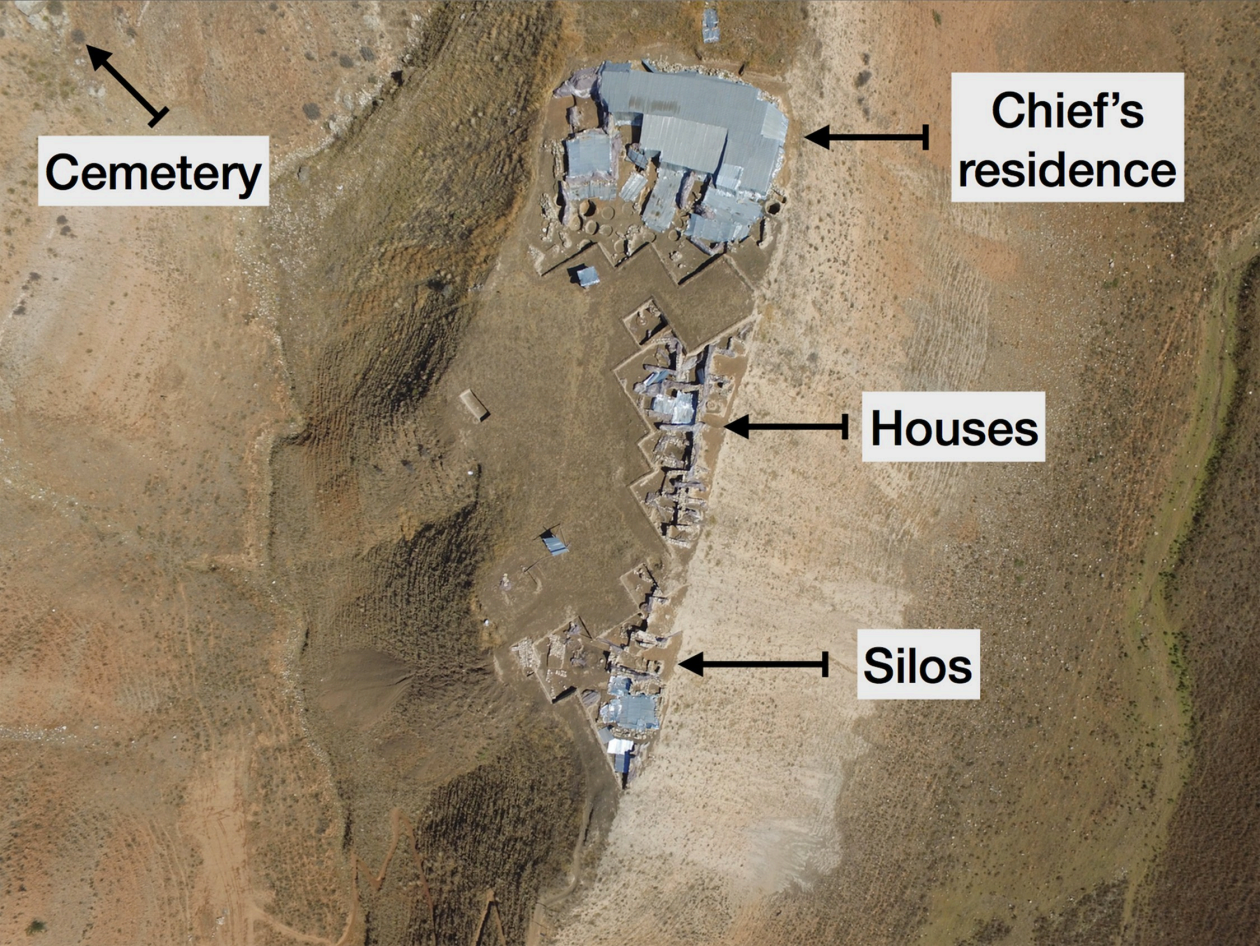
The settlement and cemetery areas of Resuloğlu (© Resuloğlu excavations archive).

Resuloğlu is located in the Delice Valley, west of Çorum (**Figs 1 and S1**). The site is situated on a hilltop, unlike the traditional Anatolian settlements, which are located on mounds or flat land sites. It overlooks the Delice Valley from Kavşut, in the south, to Kula, where the river meets Kızılırmak, the ancient Halys [52–55]. The site consists of a cemetery area and a settlement that spreads over two opposing ridges separated by approximately 100 m today; however, at the time of habitation these ridges might have been connected (**Fig 3**).

The ceramic and material evidence unearthed at the settlement of Resuloğlu demonstrate two chronologically distinct phases (i.e., sub-phases) within the second half of the 3rd millennium BC. Radiocarbon samples collected from different rooms verify this dating (Charcoal from DK-10/101; 1σ: 2470–2290 cal. BC, 2σ: 2470–23400 cal BC; carbonized grains from room I; 1σ: 2286–2042 BC, 2σ: 2455–1975 BC; charcoal from room K; 1σ: 2473–2235 BC, 2σ: 2570–2195 BC) [86: p.195–196]. While recent unpublished radiocarbon dates from the settlement pinpoint the end of the Early Bronze III to 2200–2150 BC, the existence of Çıradere-painted and Karum III–IV type of pottery extends its chronology to 2150–2050 BC. The material corpus of the cemetery, especially ceramics, reflects the assemblage of the settlement. Therefore, the settlement and the cemetery fall chronologically into the Early Bronze II–III [53–55; 87]. During this period, the region was known as the homeland of the Hatti people, whose culture contributed to the formation of the Hittites towards the end of the Middle Bronze Age.

Discovery of both the settlement and the cemetery side-by-side makes Resuloğlu an exceptional case to study the social, cultural, and economic setting of highland communities during the latter half of the 3^rd^ millennium BC. The hilltop and strategic position of Resuloğlu provided the site access to the networks from central Anatolia to the Black Sea region. The connection of the site to the exchange routes is evident through the burial goods made of various media (metals, semi-precious stones, vitreous materials) in its cemetery area.

The large number of metal artifacts used as burial gifts were largely recovered in pithos burials, as well as in a small number of simple and stone lined graves [54: p.15]. While the cemetery displayed a large number of metal artifacts, metal was a rare commodity at the settlement. Regardless of its wealth in metals, no evidence of mining or metallurgy like slags, crucibles, molds, or tuyeres has been identified, demonstrating the dearth of any metallurgical practices at the site. Settlers of Resuloğlu must have been obtaining certain metal objects via exchange networks through a direct or indirect reciprocal relationship with a so-far unidentified mining community/communities. They must have been contributing to this give-and-take system with grain, as demonstrated by excavations in the settlement area, which unearthed more than 60 silos dug into the ground, each with a capacity of almost a ton [87].

Delice Valley, where Resuloğlu is located, was a part of long-distance trade network after 4500 cal BP supporting the emergence of social, political, and economic complexity [87: p.571]. It was a settlement for a small agrarian community exchanging goods in the hills and plains outside of the highlands. Finds indicating links between these areas and the population of Resuloğlu include carnelian, obsidian, flint, and a small number of imported pottery pieces, which is otherwise local and handmade. Research conducted further north of Resuloğlu towards Paphlagonia confirmed a similar system associated with natural resources including obsidian, flint, and rock-salt [88], [for a similar case in a mining community see also 89: p.191]. Resuloğlu, like its contemporaries in the region, was fortified and in a defensible position, which may indicate a threat of raiding. R. Matthews [88: p.32] suggested possible competition over raw materials, as well as arable land, which is inadequate in the region. Resuloğlu had accessed such arable parcels, as verified by the notable number and size of silos.

T. Yıldırım [52–54], the site’s excavation director, suggests a house complex with more than 15 rooms, silos, and a separate enclosure wall unearthed at the settlement belonged to a ‘feudal lord’. This definition can be equated to the term ‘chief’, hierarchically locating the leader above tribal and below state rulers [90]. Evidence for a population of approximately 100–150 people has been discovered, strongly supporting the argument that Resuloğlu was a simple chiefdom [87; 91]. The chief must have had control over production and exchange of goods [92: p.4–10], thus achieving a certain degree of power and wealth and presenting a reasonable explanation for the fifteen-room house complex with an occupation area of 100 x 50 m at this settlement. Limited size of arable lands and semi-isolated locations at Delice Valley are suitable for chiefdoms. These features of the landscape make the region less favorable for centralized systems like kingdoms or imperial systems, which require larger landmasses for agropastoral production and accessibility [87: p.587].

Resuloğlu presents a north-central Anatolian prototype for highland chiefdoms, for which the scale and organization of production, supply of raw materials, possible use of local resources, as well as the extent of exchange require further exploration. S. Shennan [89] described such communities as autonomous, without a monopoly of any single group in production and exchange, within which raw materials functioned as proto-currency. The raw materials could have been supplied from neighboring regions, making the control over common and accessible materials an unimportant parameter for power differentials or inequality among similar communities [93]. Shennan [89: p.91] argued that small autonomous settlements were abandoned at one point, and their means of production may have come under centralized control, based on a case study of the copper production in the 2^nd^ millennium BC mining communities in the Alps. This hypothesis is supported by Resuloğlu, which was abandoned at the end of the 3^rd^ or the beginning of the 2^nd^ millennium BC, when the social organization of most Anatolian communities was evolving into a centralized system.

### Antimony-bearing artifacts in Resuloğlu: Methodology and results

While the archaeometric investigations of the complete metal assemblage of Resuloğlu are on-going, and partially published elsewhere [51; 94–96], this paper presents the compositional results of 12 metal artifacts, out of a total of 309, making up 3.9% of the whole corpus. This low percentage in the corpus indicates the rarity of antimony alloys. Aside from metal objects, including a ring, three earrings, six pins, and two beads, this paper introduces investigation of a piece of antimony–arsenic ore recovered in the settlement. All necessary permits were obtained for the described study, which complied with all relevant regulations. All analyses are conducted in the Çorum Museum (Çorum, Turkey), where the material subjected to this study are kept. The legal permits are issued by the Çorum Museum and the Turkish Ministry of Culture and Tourism (Ankara).

For the pXRF analysis, a portable Bruker Tracer SD-IV was used. The X-ray tube type used was a rhodium target. 40kV and 15.80μA were selected under air (non-vacuum), with a titanium-aluminum filter of 25μ titanium in layer 1 and 300μ aluminum in layer 2. This is the automatic filter specification of the instrument in filter number one and is the standard setting of the instrument used for metal analysis. The instrument was calibrated according to copper alloy standards BCR 691 A, C, D, and BAM 211. Analysis time was adjusted to 180 seconds. Each reading was repeated three times to obtain an average. For some artefacts such as pins, we selected at least two different points for analysis; one from the shaft and one from the head in order to examine any possible variation in the alloy type and/or composition between different parts of the same object. This protocol is in concordance with previous research [97: p.83–84].

Analyses were conducted on a stand in order to minimize any instability caused by vibration of the instrument. It should be mentioned here that especially antimony like tin has a tendency to be enriched on the surface of copper objects on corrosion due to geochemical immobility. Thus, all surfaces of the artifacts were mechanically cleaned in the Çorum Museum or in collaboration with the central restoration unit of the Anatolian Civilizations Museum, Ankara; severe corrosion was not visible on the surfaces. For pXRF analysis, the size of the surface to be analyzed should accord with the window of the instrument’s detector; this prevents measurement of a void and/or background, and thus provides more accurate results. For the Resuloğlu assemblage, the instrument window was indeed filled with the surface to be analyzed; thus distortion in the results is unlikely. The results are listed in **Table 4**. The statistical evaluations and the graphs are prepared by using the software JMP SW 14.

**Table 4:** Elemental compositions of EBA antimony-bearing artifacts from Anatolia discussed in the text. All compositions are listed in %wt. All Resuloğlu objects are analyzed with pXRF (nd: not determined; -: no available data; *: μg/g). For the reference of other analytical data and methodology see: Ahlatlıbel [42: p.121, 177]; Polatlı [42: p.141, 171]; Resuloğlu [51]; [for sample Ro-05-M94, 94: p.96, fig.5; 129: fig.6]; Alaca Höyük [56]; Kültepe [5,7: p.199, table 1]; Tarsus-Gözlükule [3,2: p.202, table 4]; Soloi [59: p.201]; Troas [42: p.143, 172]; Tepecik [98: table 3]; Arslantepe [33]; Troy [4,5: p.575, 578–579, tables 3, 4].

Site	Excavation or Museum Inventory number	Object type	Alloy type	Cu	Sn	As	Sb	Pb	Ni	Co	Fe	Ag	Au
Resuloğlu	18_1_2007/8614–1	Ring	Cu-Ag-Au-Sb	27.3	nd	nd	4.22	nd	nd	nd	nd	51.96	16.6
Resuloğlu	Ro.07_Etd.10/ Etd. 1088–2	Earring	Cu-As-Sb	96.37	nd	1.57	2.2	0.05	0.15	nd	0.08	0.09	0.01
Resuloğlu	Ro.10_1/ Etd. 1260	Pin	Cu-As-Sb-Fe (?)	91.02	0.01	1.76	1.45	0.02	nd	0.06	4.8	0.09	0.03
Resuloğlu	Ro.11_8/9104	Pin	Cu-Sb	96.27	nd	0.24	2.05	0.15	0.01	0.03	0.73	nd	nd
Resuloğlu	Etd. 1243	Pin	Cu-Sb	95.5	0.03	0.9	1.19	0.16	0.01	0.03	1.74	0.05	nd
Resuloğlu	Etd. 1244	Pin	Cu-Sb	98.26	0.25	0.02	1.06	0.2	nd	0.02	0.27	0.05	nd
Resuloğlu	Ro.10_6/ Etd. 1248	Pin	Cu-Sb	95.74	0.78	0.73	1.1	0.65	nd	0.03	0.73	0.03	0,01
Resuloğlu	Ro.11_8/ Etd. 1276	Pin	Cu-Sb	97.39	0.01	0.35	1.22	0.01	nd	0.01	0.57	0.08	0.01
Resuloğlu	Ro.11_30/ Etd. 1305–1	Earring	Cu-Sb	94.64	0.94	0.05	2.5	0.55	nd	0.04	nd	0.16	0.03
Resuloğlu	Ro.07_Etd18/Etd 1096–3	Bead	Cu-Sb	96.29	nd	0.86	1.44	0.09	0.17	nd	0.17	0.22	0.18
Resuloğlu	Ro.11_30/Etd.1305–2	Earring	Cu-Sn-Sb	89.18	1.45	0.03	5.77	1.12	nd	0.04		0.43	0.13
Resuloğlu	Ro-05-M94	Bead	Cu-Sn-Sb	79.8	16.8	0.4	2.13	0.16	nd	nd	nd	nd	nd
Ahlatlıbel	–	Bead	Cu-Sb	-	-	0	5	-	-	-	-	0.04	-
Polatlı	–	Vase frag.	Cu-Sn-Sb	-	5	0.2	5.2	-	-	-	-	0.04	-
Alaca Höyük	7027	Figurine_head	Cu-Sn-Sb	80.8	10.4	-	6.9	1.5	-	-	-	-	-
Alaca Höyük	7027	Figurine_belly	Cu-Sn-Sb	84.2	8.8	-	5.5	1.5	-	-	-	-	-
Kültepe	Kt11-238B	Pin	Cu-As-Sb	97	-	1.13	1.34	1.21	-	-	-	-	-
Gözlükule	T47-87	Seal	Cu-Sb	86.5	-	-	10.3	-	-	-	-	0.07	-
Gözlükule	T47-1	Seal	Cu-Sb	86.2	-	-	13.8	-	-	-	-		-
Soloi	3400a	Dagger	Cu-Sb	94.45	0.05	-	2.5	trace	-	-	-	-	-
Soloi	3393	Seal	Cu-Sb	<96.5	0.5	-	2	>>1	-	-	-	-	-
Troas	3798	Vase frag.	Cu-Sn-Sb	-	9.1	0.88	1.35	-	-	-	-	-	-
Tepecik	T.69-922	Pin	Cu-Sb	86.71	-	0.8	1.8	-	-	-	-	0.04	-
Arslantepe	ARSL 58	Beaker	Cu-As-Sb-Fe (?)	76	-	8.66	2.74	0.44	0.17	-	4.6	0.4	-
Troy	HDM 253	Axe	Cu-Sb	98	<0.013	0.57	1.24	0.00009	0.0247	<3*	<0.15	2.6*	0.8*
Troy	HDM 267	Vase frag	Cu-As-Sb	92	0.016	1.4	1.67	0	0.38	<7*	<0.4	8300*	1.4*

The pXRF analyses show a variation in the use of antimony-bearing artifacts, even in a moderate sample set. Chemical compositions are treated as decisive for copper alloy types. Elemental composition of greater than 1 wt% is accepted as an alloy (antimony-bearing artifact). Lead is as an exception for which the limit is set as 5 wt%. These limits are used here are in concordance with similar studies [e.g., 57], hence allowing us to present a common ground for comparisons. All percentages referred here are given wt%.

**Fig 4:**
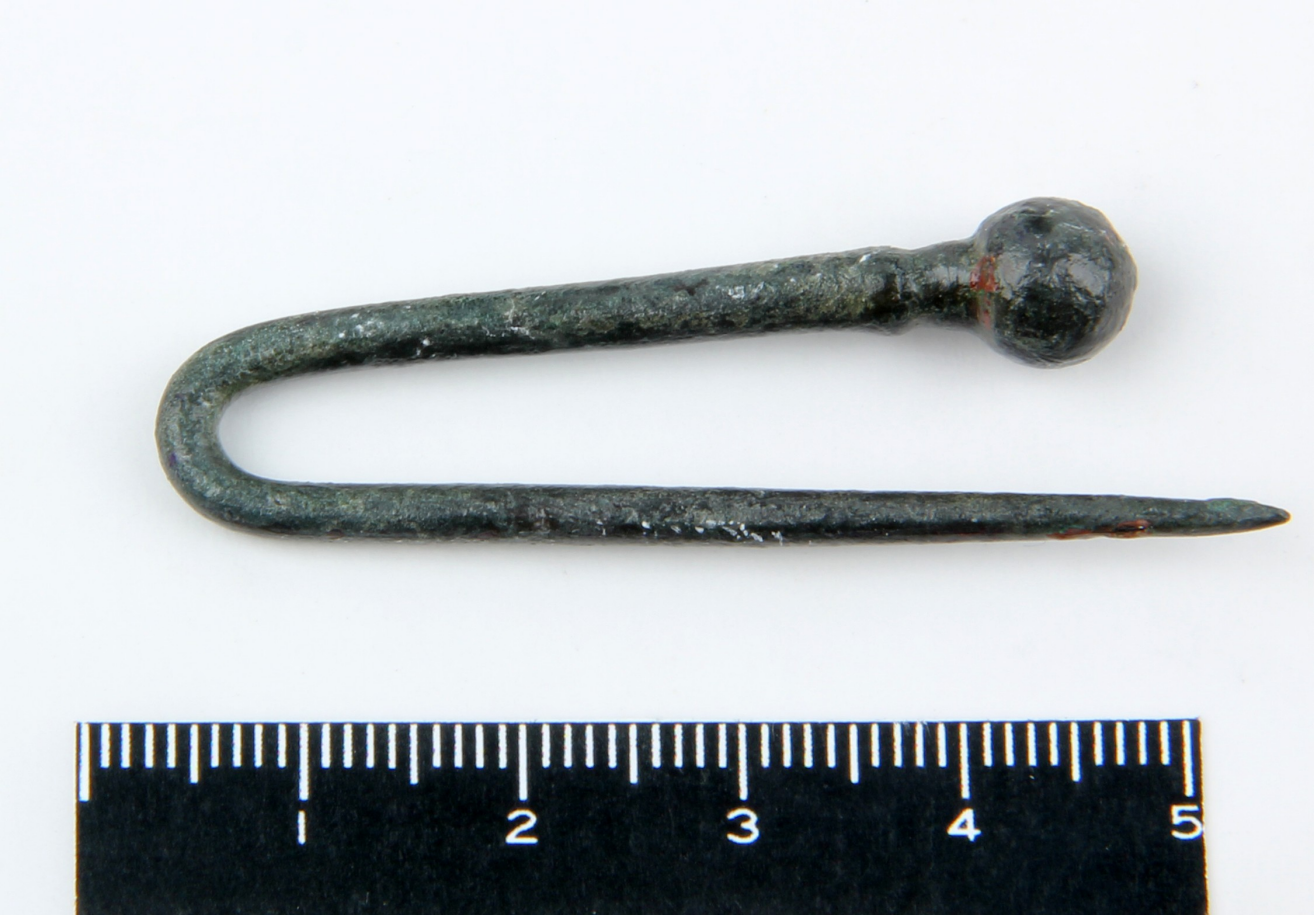
The antimonial copper pin from Resuloğlu with inventory number 9104 (© Resuloğlu excavations archive).

**Fig 5:**
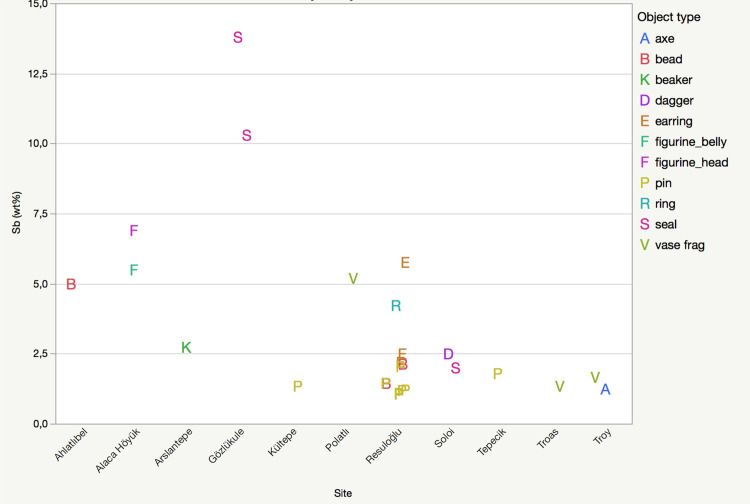
Antimony contents in the Early Bronze Age artifacts discussed in the text (© Resuloğlu excavations archive).

Four different types of antimony-containing alloys were found: copper–antimony (Cu-Sb), copper–arsenic–antimony (Cu-As-Sb), copper–tin–antimony (Cu-Sn-Sb), and copper–silver–gold–antimony (Cu-Au-Ag-Sb) (**Fig 2**). Copper–arsenic–antimony–iron (Cu-As-Sb-Fe (?)) is shown seperately in **Fig 2**to accentuate the high iron contents of two samples. The Cu-Sb alloy dominates this sample set; antimony amounts vary between 1–4%. Five out of six pins (museum no’s: 9104, Etd. 1243, Etd. 1244, Etd. 1248, Etd. 1276) are antimonial copper, with the highest amount of antimony detected at 2.05% (**Fig 4**). The other Cu-Sb alloy objects are an earring and a bead with inventory numbers Etd. 1305–1 and Etd. 1096–3, respectively. The earring contains 2.5% antimony with a notable tin content of 0.94%. The bead includes 1.44% antimony and 0.86% arsenic (**Fig 5**).

**Fig 6:**
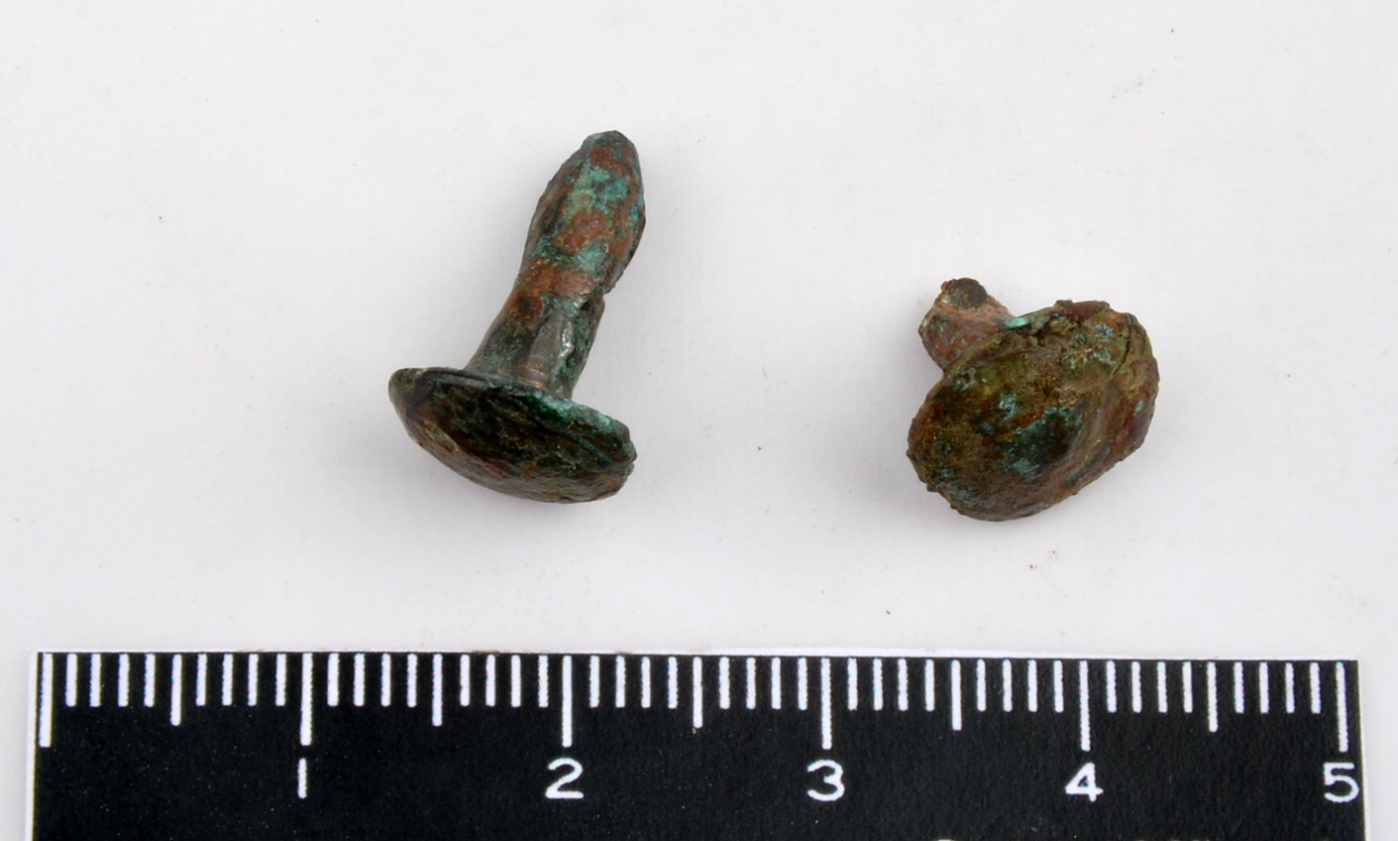
The Cu-As-Sb earring from Resuloğlu with inventory number Etd.1088. The complete earring is analyzed and classified as Etd.1088–2 (© Resuloğlu excavations archive).

**Fig 7:**
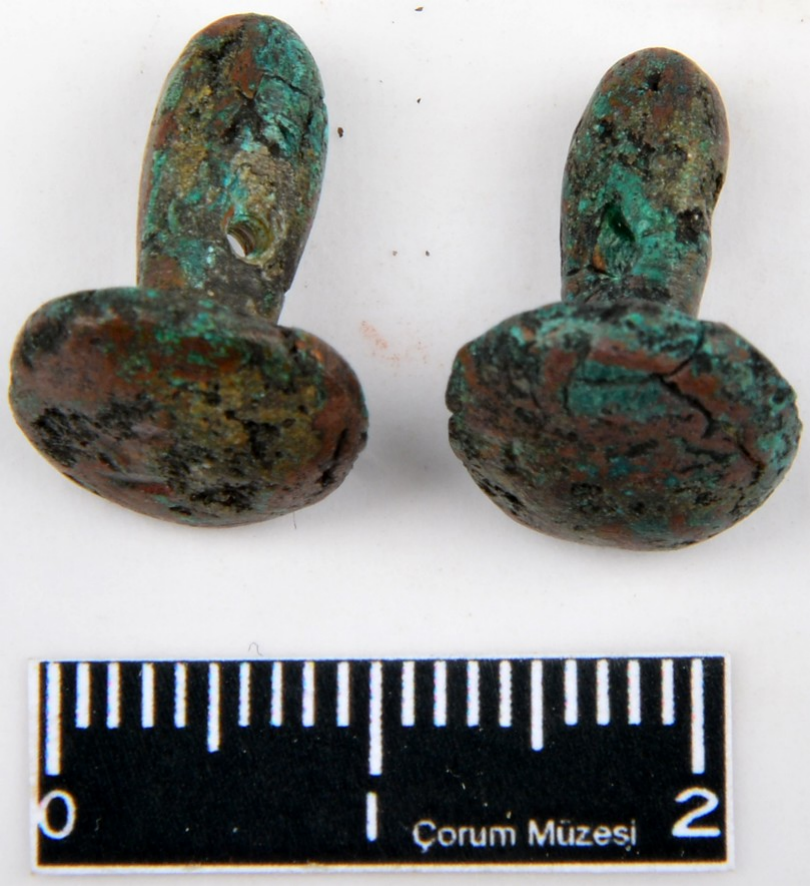
The Cu-Sn-Sb earring from Resuloğlu with inventory number Etd.1305. The earring on the left is analyzed and classified as Etd.1305–2 (© Resuloğlu excavations archive).

There is one Cu-As-Sb object: an earring. The earring, Etd.1088–2, contains 1.57% arsenic and 2.2% antimony (**Fig 6**). Another Cu-As-Sb with a high concentration of iron (4.8%) is the pin, Etd. 1260. It comprises 1.76% arsenic and 1.45% antimony, and 4.8% iron. In **Fig 2**, it is classified as Cu-As-Sb-Fe(?) to differentiate its high iron content, which shows similarities in composition with the Arslantepe beaker. Among the Cu-Sn-Sb objects, a bead (inventory no: Ro–05–M94) contains 16.8% tin and 2.13% antimony [94; 95]. This bead is the most tin-bearing ornament in this assemblage. However, it should be noted that Cu-Sn objects dominate Resuloğlu’s metal assemblage, among which beads have tin concentrations as high as 18–20% (**S2 Fig**). The other object is an earring with 5.77% antimony (**Fig 7**). This is also the highest antimony content in the assemblage. The Cu-Ag-Au-Sb quaternary alloy (?) observed in ring 8614–ring–1 is unique not only for the site but also among other Early Bronze Age sites [51: fig. 4].

Previous studies at Resuloğlu covered pXRF analysis on a small group of selected metals [95; 96; 99]. The selection is mostly based on the identification of ‘exotic alloying practices’ at the cemetery; some of these results are worth discussing. pXRF analysis of a mace head from Resuloğlu (9-17-2006) first published as 99.9% copper [96], but later suggested to contain 18.1% antimony, 1.16% arsenic, and 80.3% copper [99: p.254, table 1]. While the discrepancy between these two publications is unclear, a recent analysis of the mace head confirms it as ca. 99% copper [51: p.150, fn. 50]. A pin (9-8-2006) suggested to be made of 2.64% antimony, 2.41% arsenic, and 95.2% copper [99: p.254, table 1] was reanalyzed by Dardeniz and found as arsenical copper with ca. 0.35% antimony. The complete publication of the Resuloğlu metals will discuss this issue in detail.

To sensibly discuss the antimony-bearing alloys in Resuloğlu, the antimony content of the rest of the metals from the site should be introduced. At Resuloğlu, a total of 309 metals have been analyzed systematically with the pXRF. This data set is so-far the biggest data set collected from a single site dating to this period in Anatolia. With this backdrop of information, the most common alloy detected at Resuloğlu is copper–tin (Cu-Sn), followed by copper–arsenic (Cu-As), and unalloyed copper (Cu). While the complete compositional results are in preparation for final publication, only 80 objects contain over 0.01% antimony within the collection (**S3 Fig**). At the rest of the corpus (ca. 75%), antimony has not been detected. The mean value of antimony among the 80 samples is calculated as 0.42, signaling objects with more than 1% antimony as significant to contextualize within the metal collection of the site.

Out of 12 antimony-bearing artifacts, only two (inventory numbers 9104, Etd. 1276) were found in the settlement, where a total of 17 metal objects have been documented so far. Considering the low number of metal objects uncovered at the settlement, these two artifacts represent 10% of the settlement’s assemblage. The majority of metals uncovered at the settlement were made out of copper (ten pieces), followed by five arsenical copper objects. Metal objects uncovered at the settlement are mostly utilitarian, while the rest of the tools found at the settlement are made of stone.

### Sources of antimony

The minerals and ore deposits from which antimony and/or arsenic bearing alloys could be produced are among the less studied topics of ancient metallurgy. Antimony is present only in very low concentration in native copper. 1,065 native copper specimens were examined through neutron activation analysis and show less than 0.01% antimony, with a maximum of 0.089% and a mean value of 4.4 ppm [100: p.23, table 2.2]. Thus, the antimony in native copper cannot result in a copper–antimony alloy. It suggests that antimony minerals, or antimony-bearing minerals, were used, deliberately or accidentally, to obtain antimony or antimonial copper objects.

T. A. Wertime noted [101: p.1257] ‘long and unintentional trials with impurities such as arsenic and antimony’, and proposed smelting of sulfide ores giving rise to Cu-As-Sb alloys. Even though Wertime’s ‘standard model’ for metallurgy no longer effective, scholars agree that unintentional alloying is possible through antimony and/or arsenic bearing copper ores, or such ores could have been added to molten copper to produce alloys [5: p.40; 102: p.485].

The relationship of trace elements and antimony signals the source of antimony. A positive correlation between antimony and arsenic, bismuth and silver in antimony-rich objects demonstrates the utilization of fahlerz type ores. If native antimony and arsenic, realgar (AsS), orpiment (As_2_S_3_), arsenopyrite (FeAsS), or stibnite (Sb_2_S_3_) had been used as the source material, a correlation between antimony and arsenic would not be plausible [13: p.131]. At Resuloğlu, while two objects show fahlerz signal, any correlation between antimony and arsenic, bismuth or silver is not observed (**S4 Fig**). This suggests that physical mixtures of copper (not necessarily native copper) and antimony–arsenic–rich ores could have been melted together at Resuloğlu intentionally. These ores might occur together in the same or adjacent deposits; or they might have been deliberately collected from two different deposits and mixed.

**Fig 8:**
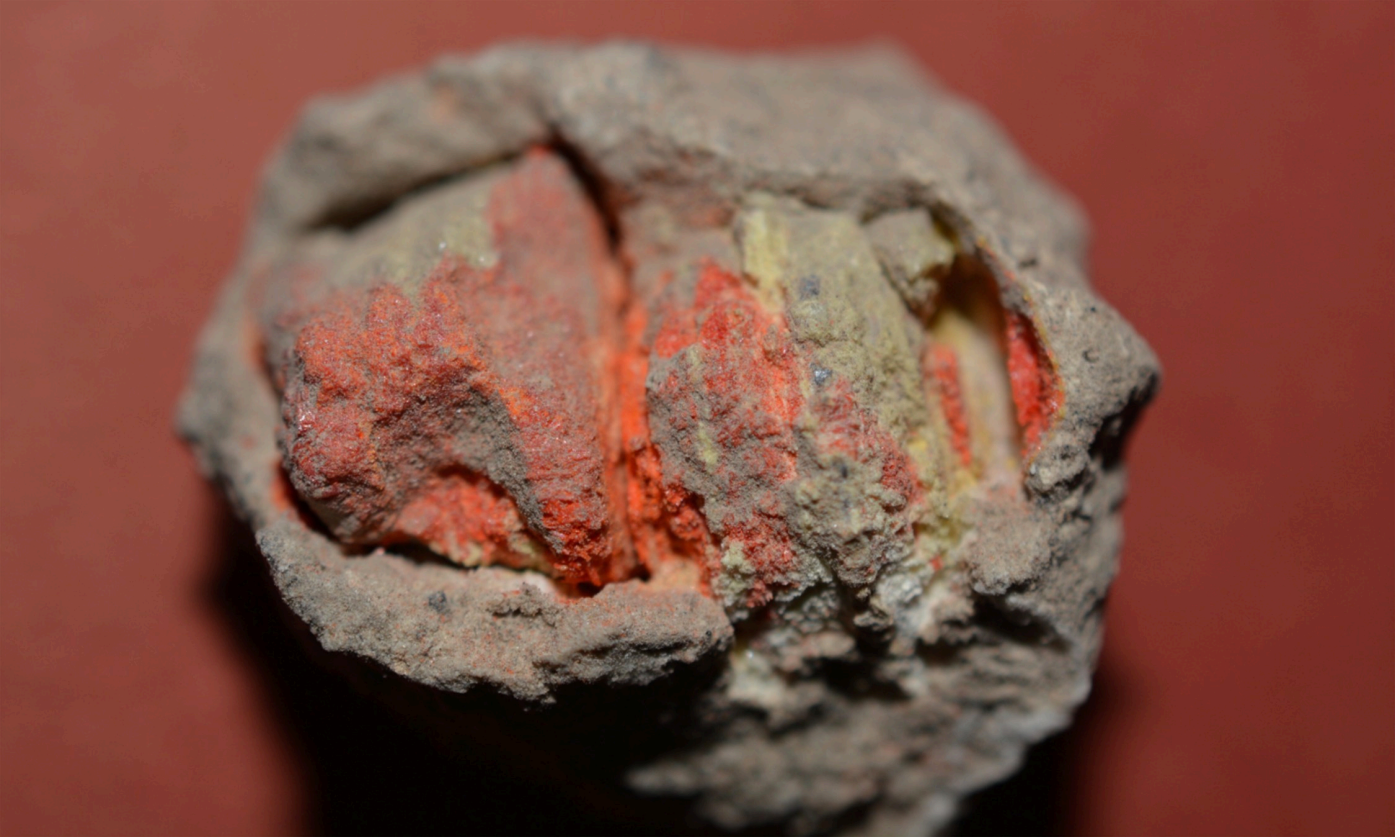
Orpiment–realgar ore fragment found in association with stibnite at the southern edge of Resuloğlu (square ZF 46), not to scale (© Resuloğlu excavations archive).

**Fig 9:**
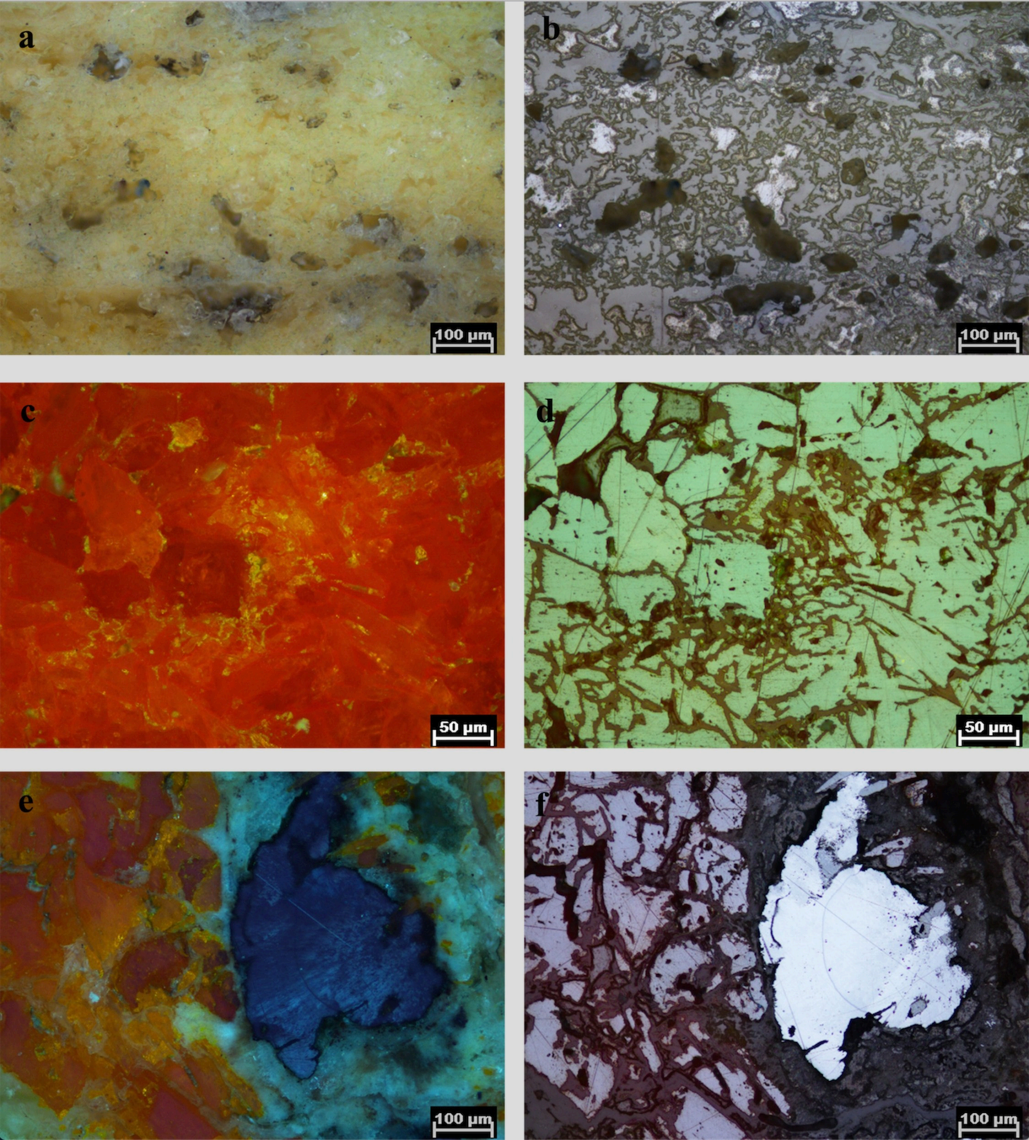
Thin section petrography of the ore fragment. **a)** orpiment, single Nicol; **b)** orpiment, crossed-Nicols; **c)** realgar, single Nicol; **d)** realgar, crossed-Nicols; **e)** orpiment–realgar with stibnite, single Nicol; f) orpiment–realgar with stibnite, crossed-Nicols.

A piece of ore recovered at the settlement of Resuloğlu implies recognition of antimony–arsenic ores and supports the idea of melting physical mixtures of copper and antimony–arsenic–rich ores together to produce some of the antimony-bearing alloys. Even though Resuloğlu has not yielded any evidence of metallurgical practices, an ore fragment found in square ZF 46 at the southern edge of the settlement provides sufficient evidence for the argument that antimony–arsenic-rich ores were known and collected by the settlers (**Fig 8**). The yellow- and orange-colored mineral lump was found in 2016 and is coated with a thin crust. It consists of two fragments, both of which were approximately 2–3 cm in size. An on-site examination of the ore with pXRF confirmed the existence of arsenic and antimony in the pieces (**S5 Fig**). A polished cross section of the sample was examined under a Nikon LV100 Pol research microscope. Based on polarization colors and pleochroism–anisotropism characteristics the ore is identified as an orpiment (**Fig 9A and 9B**)–realgar (**Fig 9C and 9D**) mineralization associated with stibnite (**Fig 9E and 9F**). Use of this mineral might well result Cu–As–Sb alloy, documented both in Resuloğlu and Kültepe corpora. At Durağan (Black Sea), similar orpiment–realgar mineralization were documented with arsenic contents as high as 55.9% [103: table 2]. Eight ore samples collected from the Durağan orpiment–realgar mineralization yielded varying amounts of antimony of 0.03–0.11 percent [103: table 2].

Finding the orpiment–realgar ore fragment associated with stibnite at Resuloğlu demonstrates that the settlers likely intentionally collected or traded antimony–arsenic rich ores. The distinct color of the ore could have been one of the reasons for its collection. Yet, this does not necessarily suggest the recognition of antimony as a distinct material during the Early Bronze Age. The utilization of such ores at Resuloğlu, however, signifies access to the mineral resources in the north via trade routes, as suggested based also on the typological similarities of metal and ceramic assemblages [31; 55]. While ores have been also used as pigments, no evidence of pigment use has been uncovered at Resuloğlu. Over two decades of excavations have not yielded any other ore fragment. While being plausible, any indication of the use antimony-bearing arsenic ores as a pigment in Resuloğlu has not been detected so far.

The ore fragment adds evidence to the argument of exchange of raw materials [88] including arsenic-bearing ores within central Black Sea networks from the northernmost frontier of the central Anatolia. North-central Anatolia represents a topographically and ecologically diverse environment [104]. The region presents a combination of watered plains and mountainous zones, providing people with resources of the staple economy and mineral and forestry reserves. Black Sea ore studies have confirmed the presence of ore deposits in the region [105: p.82 with references cited therein]. The most promising and well-known arsenic-rich reserves in Anatolia are located in Durağan, Tavşan Dağ, Peynir Çayı, and Bakırçay (Sinop) at the Black Sea. Durağan is approximately 300 km north of Resuloğlu via the metal-rich route of Merzifon. Geological, mineralogical, and chemical investigation of the resources in the area have been initiated to understand the ancient metallurgy of the Black Sea, as well as to contextualize the arsenical copper objects found at the site of İkiztepe [24; 25; 44; 103; 105–111]. Özbal et al. [103] recorded orpiment–realgar mineralization as veins in schist–calc formations in Durağan close to Alaçam, though evidence of ancient mining was not be detected. The Black Sea ores contain certain amounts of antimony. Sayre et al. [105: table 8] noted Merzifon (lead slag) and Giresun/Tirebolu (galena, galena–sphalerite, or sphalerite) including as high as 2.66% antimony, along with arsenic, lead, and copper.

Other than in the Black Sea reserves, antimony is generally found in small and irregular reserves in Anatolia. The resources are generally related to magmatic rocks like granite, diorite, monzonite, and quartz veins. Antimony sometimes occurs natively, though it is mostly found in association with pyrite, galena, and arsenic minerals, as well as with copper, lead, zinc sulfides, and quartz–gold veins trapped in sediments. Thus, antimony ores generally contain 0.3% arsenic and 0.3% lead. Considering the location of the case site, I focus on central Anatolian deposits in and around Delice Valley and Çorum.

**Fig 10:**
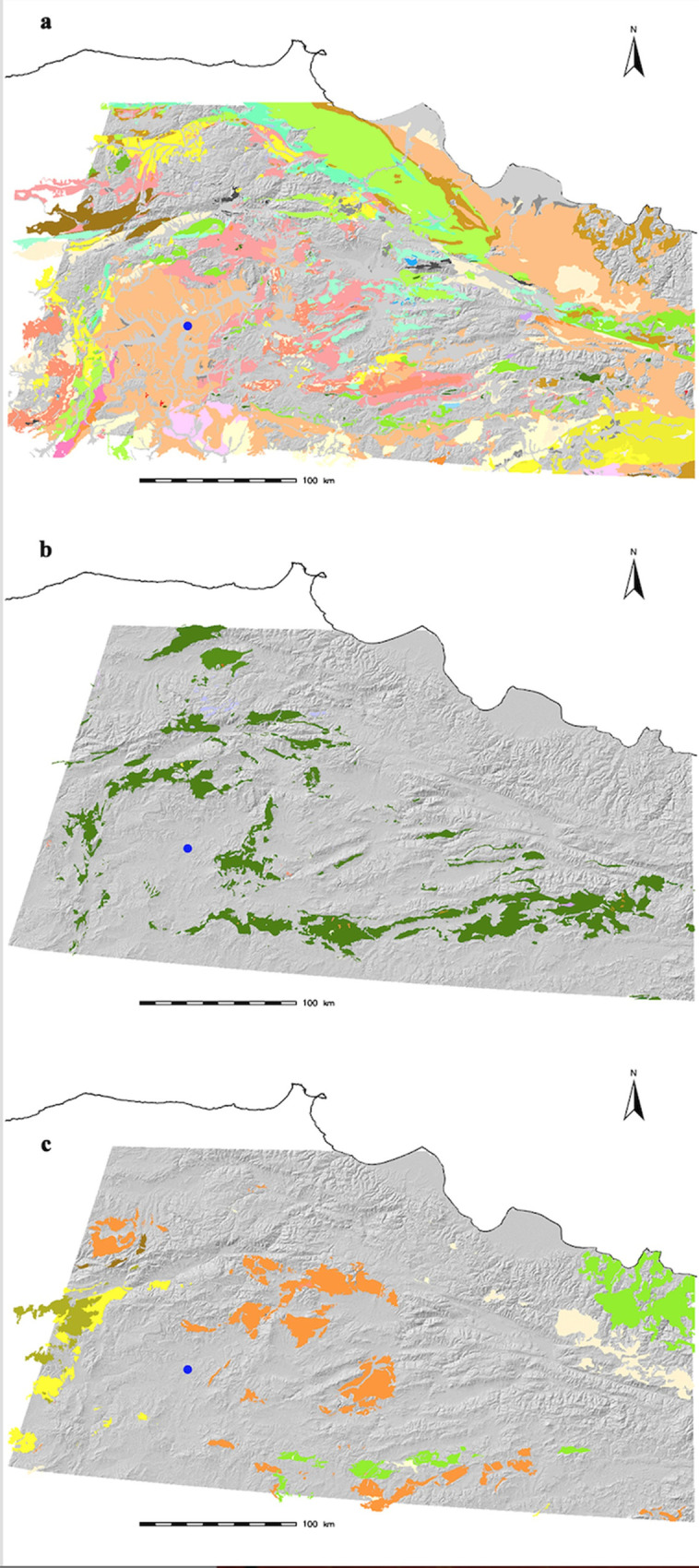
**a)** Sedimentary; **b)** Ophiolitic; **c)** Volcanic rocks in and around the Delice Valley and modern Çorum. Different colors indicate different geological ages. Blue circle indicates the location of Resuloğlu (maps: courtesy of Bülent Arıkan).

Details of the geology and geomorphology of Delice Valley are discussed in detail by Arıkan and Yıldırım [87: p.573–574]. The basin is composed of sedimentary, ophiolitic, and volcanic rocks providing suitable geology for the formation of antimony-bearing ore deposits (**Fig 10A–10C**). It is not surprising that the region is well known for its rich metallic resources, especially antimony, copper, and iron, though most of these are less important reserves for modern economic and industrial activities. Nonetheless, there is a potential of possible historic mining activities, some of which were documented in neighboring regions like Işıkdağ (Ankara), Derekutugun (Çorum), Merzifon, Tokat, and Gümüşhane [44; 106–109; 112].

The antimony reserve documented in Laçin–Obruk is known to have been exploited decades ago for eight tons of antimony, though the net reserve is unknown. Small antimony occurrences were documented in Osmancık and Tekke Köy to the north of Çorum. In Osmancık, the stibnite mineralization is associated with yellow-colored cervantite, and white-colored velentinite, and senarmontite, along with pyrite. Even though these occurrences do not have economic importance, the 1970 procurement of 7–8 tons of ore has been noted in the region [113], which might have satisfied local consumption in earlier times.

To the west of Çorum, in Ankara, lead–zinc–gold deposits containing antimony were identified near Kızılcahamam and Güven townships by Salınyaylası (Yağcıhasanlar) [114; 115: p.64]. In the village of Çukurca, stibnite sources developing in quartz–porphyry were reported in 1940 by the Turkish Geological Survey (MTA) [116]. At Işık Dağı in Kızılcahamam, located approximately 80 km north of Ankara, antimony was found in the form of arsenic-bearing boulangerite (PbS–Sb_2_S_3_) [116; 117]. This formation was once documented as yenerite [118]; yenerite is now a discredited type in the PbS–Sb_2_S_3_ group [117: p.109]. The existence of arsenopyrite in the mineralization with 44.88% arsenic and 2–3% arsenic-bearing pyrite is significant [117: p.111–112, table 1]. Relevant to the discussion here is the existence of gold and cassiterite (SnO_2_), a tin mineral, at Işık Dağı [119: p.262], which is mostly known with its major lead–zinc deposit. Işık Dağı, is located circa 100 km north of Ahlatlıbel and Polatlı, the latter of which yielded a vase fragment of Cu-Sn-Sb alloy, raising questions about the possible usage of the reserves in ancient times. While solid evidence of its exploitation during the Bronze Age is lacking, the exploitation of the mines at Işık Dağı in later periods (Byzantine times) through radiocarbon dating is confirmed [109: p.601]. Mining activities continued at the mountain in the 1800s and 1940s [120].

To the east of Çorum, the most important occurrences are found in Elalmış, Geryan, and Hacılar in Tokat–Turhal, a region still extensively exploited. The stibnite deposits with quartz gangue and sometimes pyrite and arsenopyrite are situated on both sites of Yeşilırmak, ancient Iris. Analysis of 70 tons of hand-picked ore yielded compositions of 54% antimony, 0.04% arsenic, 0.02% tin, 0.01% lead, 0.01% copper, and 0.01% zinc. With flotation, the enriched sample contained as high as 70% antimony and 1% arsenic [116; 121]. South of Tokat, in the northern part of the Zara region of Sivas, some old workings were recorded as containing antimony [115: p.65]. In Gemin Deresi (Aydın Deresi) the ore consists of stibnite and argentiferous galena in quartz. Analyses of a sample yielded 20% antimony and 194 gr per ton silver [116]. While Tokat region is rich in antimony reserves, the important Early Bronze Age site of Horoztepe at Tokat, which is contemporaneous to Resuloğlu, does not confirm antimony-bearing alloys in its metal corpus. The majority of the Horoztepe artifacts show use tin bronzes, followed by arsenical copper alloys. The results might be due to the selective study of extravagant metal objects (e.g., sun discs), or such sites might have been completely or partially integrated into different networks for metallurgical demands. Still, the available ore deposits at the region could well lead to the production of the antimony-bearing alloys.

## Discussion

The variations in the alloying practices were likely related to the supplies used and/or local conservative traditions idiosyncratic to a production zone [23: p.70]. This argument is especially valuable in Anatolia, where polymetallic ores are vastly available [122].

Almost contemporaneously to Resuloğlu, the Early Bronze Age collections of Ahlatlıbel, Alaca Höyük, Kültepe, Polatlı, Tepecik, Troas, Soloi, and Gözlükule yielded Cu-Sb, Cu-As-Sb, and Cu-Sn-Sb objects (**Fig 2**). Recognizing the sites’ chronological contrasts within the Early Bronze Age, the pin Etd.1260 from Resuloğlu and the beaker from the Early Bronze I Royal Tomb of Arslantepe differ from the rest with their exceptional iron-contents. Of these alloys, Cu-Sb is most commonly found also at Ahlatlıbel (bead), Gözlükule (seals), Soloi (seal and dagger), Troy (axe), and Tepecik (pin). Aside from Resuloğlu, Cu-As-Sb alloy is identified at Kültepe and Troy. Cu-Sn-Sb composition has been identified on different parts of the Alaca Höyük figurine and on two vase fragments from Polatlı and Troas, both of which situate on the western part of the Halys Basin. The antimony contents of these objects are generally as less than 5% at these sites, though the seals from Gözlükule have the highest amount of antimony at 14% providing an attractive sheen to the objects (**Fig 5**). The color of Cu-Sb alloys changes towards blue after the addition of 6–7wt% antimony [7]. The antimony contents indicate that the seals have appeared blue/indigo, which is even observable today [32]. Hitherto, for the majority of the rest of the artifacts discussed, it is hard to claim for a recognizable color effect of antimony.

The corpus introduced here composed of mostly pins and beads with the exceptions of three seals, a figurine, a dagger, and an axe. Seals and figurines especially align with symbolism than utilitarian use, which finds evidence at the Chalcolithic Levant. Copper alloys with high antimony and arsenic for an attractive surface color are known mostly from the region, where such alloys were used to produce elaborate and symbolic objects rather than utilitarian tools [123]. While a similar surface color is visible at Gözlükule seals, a detailed examination of Alaca Höyük figurine is necessary. The antimony levels of the dagger and the axe are below the limits of creating a color effect.

### Diffusion of Innovations (DOI) and lock-in effect

While central Anatolia enjoys abundant polymetallic resources, including antimony-bearing ores, tin bronzes dominate archaeological assemblages, particularly after the first half of the 3^rd^ millennium BC. During the 2^nd^ millennium BC, antimony alloys almost disappear from artifactual corpora, whereas written records confirm the availability and circulation of antimony most possibly in the form of its ores. Major Anatolian centers of Old Assyrian trade such as Kültepe and Acemhöyük do not yield antimony-bearing metals (>1% antimony) [e.g., 57; 124; 125]. Worth to note is the quite low number of analyzed samples from these long-excavated sites. Despite the textual data affirming antimony’s circulation, the absence of rare alloys in the archaeological record might be related to the dearth of chemical analysis. On the other hand, the lack of diversity in alloying practices and dominance of tin bronze in the Middle Bronze Age central Anatolia support the idea of shifting from diverse alloys to a more uniform pattern.

Looking from a materialistic perspective and considering the availability of resources, central Anatolian geology does not show any restriction on the exploitation and use of antimony or copper resources. Highland regions are confirmed to favor diversity [126]. The local availability of polymetallic sources in Anatolia contributed to the metallurgical experimentation and the variety of alloying practices that characterized the Anatolia highlands communities [122; 127]. The existence of different copper alloys with antimony as early as the Early Bronze Age dispel possible technological concerns about whether antimonial alloys have ever been, intentionally or unintentionally, produced either in the region or beyond. On the other hand, Anatolian evidence has gaps regarding the Early Bronze Age metal production centers; thus, no confirmation is possible at this stage about the intentionality of the production process. Similar to arsenical copper alloys, use of antimony has a problematic nature regarding the identification of intentionality. Therefore, this research does not intend to reconstruct the production of antimony-bearing alloys or claim for the intentionality of these alloys. Without archaeological data from actual production/workshop zones, it would not be realistic to propose process scenarios. This study shapes around the standing question: why did the practice of antimonial alloys not spread and become widespread despite the geologically and naturally suitable environmental backdrop of the region?

Technological competence and/or geological accessibility are not enough for producing certain materials. Social and economic contexts of communities should also be taken into account [1]. Using the frame of diversity of metallurgical practices, I approach the topic through the discourse of invention and innovation.

Technological phases, based on technological process, are proposed to track the discovery, use, trade, circulation, and discard of materials. Starting with the ‘discovery’ of the material (e.g., alloy, object type), the technological process continues to the ‘invention’ (or ‘application’) stage, when the discovery finds a potential use. Archaeologically, invention is commonly used to define the intentional or unintentional discovery of a new idea, material or process. Invention might result in a completely new product or could be a combination of already-existing technologies to obtain a different final-product [128–131]. It is not easy to archaeologically pinpoint the emergence of ideas, which has been addressed both in the explanations of invention and innovation. While invention and innovation have interwoven and ever-shifting definitions in archaeological context [128; 132], the discovery and invention stages are followed by the ‘innovation’ (‘diffusion’) stage, referring to the spread of the invention in its region and beyond [132–134].

Technological processes do not always progress linearly along these stages. A discovery might get forgotten or has not passed to the invention stage. A. J. Shortland [134] demonstrated the utility of this approach with an examination of Bronze Age glass technology, which shows a gap of approximately a thousand years between its discovery somewhere in northern Syria/Mesopotamia at ca. 2500 BC, and its dissemination around 1500 BC in the Near East. For an invention to become popular, i.e., to spread within a society and abroad, a critical mass in the society needs to adopt it, whether for technological or cultural reasons. This theoretical approach is known as the diffusion of innovations theory (DOI).

E. M. Rogers, who developed the DOI theory [133], identified certain parameters requiring a community to adopt innovations like new ideas, technology, materials, or objects. These parameters are: a) *relative advantage*: the degree of being better than the one it supersedes; b) *compatibility*: agreeableness to standing needs and values; c) *complexity*: the ease of use and understanding; d) *triability*: the extend of being able to test or experiment with the innovation; e) *observability*: the detection of distinct results or benefits of the innovation. Once these parameters are established, a product or an idea diffuses through a community. Certainly, environment plays a decisive role for a discovery to flourish and pass to subsequent phases, or to get muted at an early stage [134: p.10; 135].

The archaeological evidence presented here does not demonstrate the widespread usage of antimony in alloying practices, meaning it did not pass to the innovation stage during the Bronze Age in Anatolia. Even though antimony-bearing alloys got manufactured and raw materials were accessible, it has not been popular as tin bronzes had. According to DOI, a critical mass in the population demanding antimony-bearing alloys was never achieved. While explaining the reasons are not an easy task, any the parameters of DOI might have played a role in renouncement of antimonial alloys. Additionally, after the Early Bronze Age, its use was discontinued, following a period when certain types of alloying traditions were preserved, and diversity was lacking. K. Kristiansen [136] emphasized the importance of social context to understanding the dynamics of technological processes and resistance to change. Thus, there is another level of complexity in adopting or resisting the practice of using diverse alloys, which I explain with the socioeconomic transformation towards a more centralized system in Anatolia, causing a ‘lock-in effect’. From an economic point of view, ‘lock-in effect’ defines an increase of costs over time required for exiting an established/long-run system to switch to a new system [137: p.168].

Anatolia witnessed a transformation at the end of the 3^rd^/ at the beginning of the 2^nd^ millennium BC. When intensified trading activities occurred in the Old Assyrian Trading Period (ca. 1970/1950–1750/1700 BC), powerful economic institutions developed within the system to control the network and benefit from the trade. When these profitable administrations have been established from Anatolia to Aššur, it might have been difficult to shift to another economic strategy from the perspective of maintaining costs. Therefore, the economy of the centralized city-states could have been ‘locked-in’, which includes pushing certain raw materials of trade, like tin, into the system, which, in turn, causes use of local variants to diminish. I suggest that this also prevents formation of a critical mass of demanders or adopters in the community for ‘out-of-business’ goods and pushes them to what is easily available in the market. The subsequent Hittite period fits in the same scenario. The centralized imperial system favors power over economy, which requires control over trade and market economy, causing economic intensification to increase and diversity to decrease.

## Conclusions

Anatolia is rich in polymetallic resources, which enabled ancient communities to produce alloys experimentally or unintentionally. The availability and accessibility of minor deposits, most of which are unknown to modern scholars, should have sufficed for small communities to exploit and use to manufacture objects needed for common use or for burial gifts. The use of local minor reserves along with major sources must have served not only the metal technologies and traditions of the archaeological settlements in the source’s vicinity and abroad, but also as a means for direct or indirect relations within a give-and-take system. Regardless of the very limited mining and production evidence compared to the artifactual assemblages in Anatolia, the interaction of ancient societies with their environment sourcing raw materials is detectable through the diversity in alloying practices as exemplified here with antimony.

The earliest occurrence of antimony-bearing alloys in Anatolia dates back to the 3^rd^ millennium BC. Examples have been recovered not only in major sites like Alaca Höyük, Kültepe, and Gözlükule but also in small and agrarian communities. Resuloğlu dates to the latter part of the 3^rd^ millennium BC and presents a significant example of such settlement patterns. Possibly acting autonomously, without the monopoly of any single group on production and exchange, raw materials including metals, stones, grain, and salt must have functioned as a proto-currency. Common and accessible raw materials could have been supplied from the neighboring regions, which must have been providing the system with grain in return for metals and semi-precious stones like carnelian and obsidian. This reciprocal system must have shaped procurement strategies, as well as the production and supply systems of artifacts manufactured through available polymetallic ores.

J. C. White and V. C. Pigott [138: p. 157] defined autonomous production units as “independent producers and suppliers and a relatively broad and flexible group of consumers.” These units must be “served by a production system that minimized production and transaction costs” [139: p.11]. Early Bronze Age communities from central Anatolia to the Black Sea coast could have enjoyed the accumulation of wealth to a certain degree, due to the accessibility of diverse metallic and non-metallic supplies, among which antimony-bearing ores and alloys play a part. Likewise, this mode of production and circulation carries implications for the centralization of power via the prompting of wealth accumulation [138: p.151]. When production and trade came under centralized control by the beginning of the 2^nd^ millennium BC, small autonomous settlements like Resuloğlu must have been abandoned, similarly to the well-argued case of the 2^nd^ millennium BC mining communities in the Alps [89].

Implementing the autonomous, non-monopolized production hypothesis in Anatolia also provides a way to explain the variety of alloying practices found during the Early Bronze Age and their discontinuation in north-central Anatolia, the site of Resuloğlu. Until a large tin trade started from Aššur to Anatolia, the Anatolian settlers must have maintained specific lifestyles in rural areas by facilitating/enjoying the diversity of their environment. The identification of different types of antimony-bearing alloys during the Early Bronze Age must be counted as a reflection of the environment that they have been exploiting and networks that such communities were in. The discontinuity of use of such alloys in the Middle Bronze Age must be the result of a centralized system with more power and control over production systems, resources, and circulation of goods. Even though antimony appears as a circulating commodity in the textual records of the Old Assyrian Trading Colonies, the centralized system molded around the powerful economic trade network, and well-established economic institutions did not carry any significant demand on antimony or its alloys. Textual records show antimony was not as profitable as tin or copper. The yet unknown reason(s) for the lack of demand likely vary from material properties to possible socio-economic variables or cultural choices. The same attitude towards antimony seems to have continued during the Hittite period, when neither the archaeological nor the textual records reveal a significant use of antimony and its alloys. I suggest that the ‘lock-in effect’ could have prevented the centralized economy of Middle and Late Bronze Age Anatolia from shifting from economic intensification to expansion, which promotes diversification.

‘Lock-in effect’ offers a way to explain discontinuity in the variety of alloy types at Central Anatolia during the beginning of the 2^nd^ millennium BC, when the region has witnessed political and economic transformations. Research on the diversity in alloying practices now demonstrates not only an alternative way of tracing patterns of production and supply systems but also serves as a possible proxy to footprint changes in organizational complexity.

## Supporting information

S1 Fig Ancient Anatolian settlements with antimony-bearing finds discussed in the text. 1: Norşuntepe (slag and ore fragments); 2: Arslantepe; 3: Resuloğlu; 4: Ahlatlıbel; 5: Polatlı; 6: Troas; 7: Gözlükule; 8: Soli; 9: Tepecik; 10: Kültepe; 11: Alaca Höyük (map created by the author).
(TIF)

S2 Fig Distribution of the Resuloğlu metal practices and their Sn contents. (TIF)

S3 Fig Distribution of the Resuloğlu metal practices and their Sb contents. (TIF)

S4 Fig Bivariation of As by Sb, showing no correlation. (TIF)

S5 Fig On-site pXRF analysis results from the ore fragment showing arsenic (As) and antimony (Sb) signals. (TIF)

S1 File Details of the pXRF analysis and objects from Resuloğlu. (XLSX)
