# Supplementary figures and images for: Why did the use of antimony-bearing alloys in Bronze Age Anatolia fall dormant after the Early Bronze Age?: A Case from Resuloğlu (Çorum, Turkey)
Source: PLoS One. 2020 Jul 16;15(7):e0234563. doi: 10.1371/journal.pone.0234563 (PMC7365396; doi:10.1371/journal.pone.0234563)

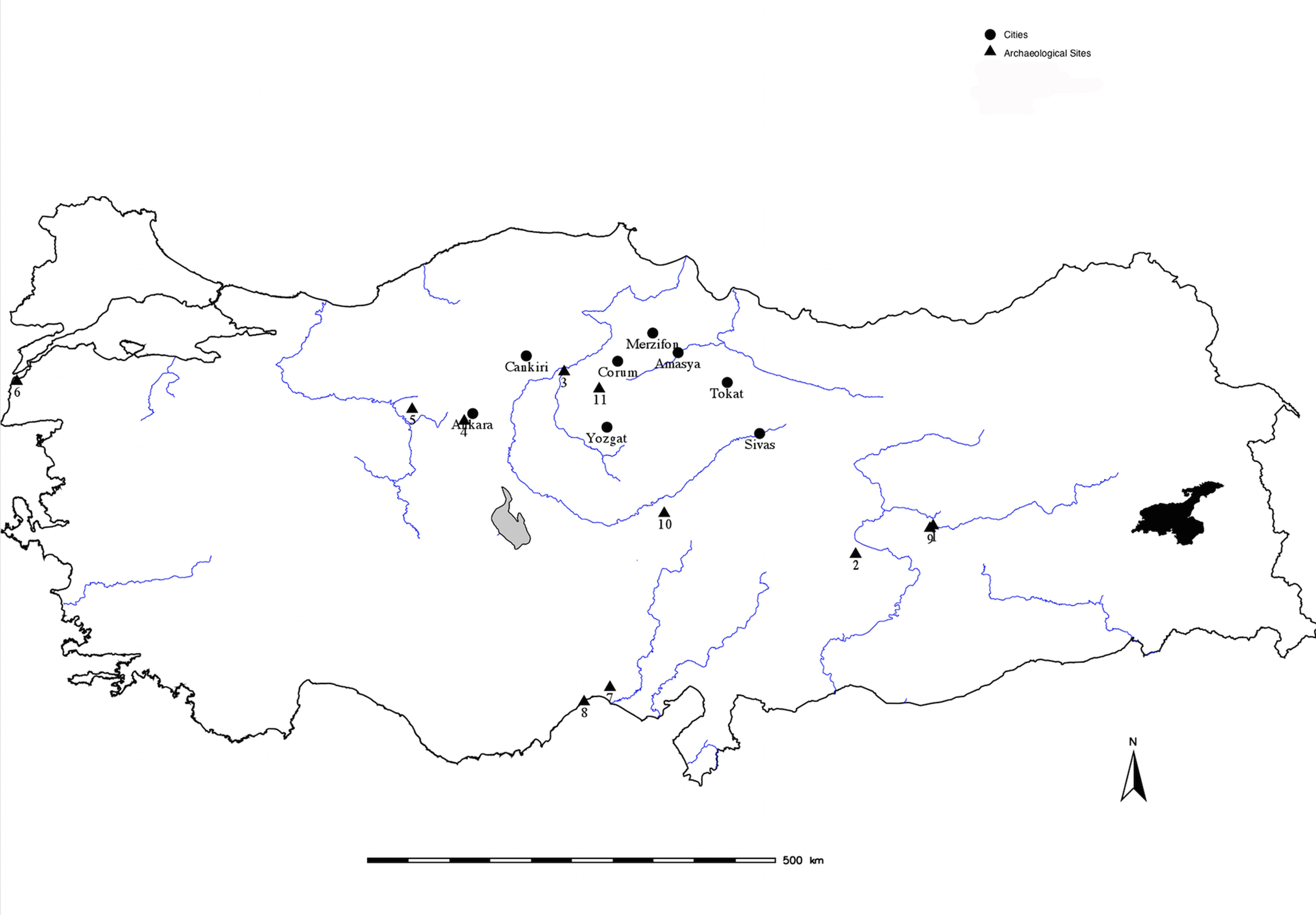

Supplement: S1 Fig — 1: Norşuntepe (slag and ore fragments); 2: Arslantepe; 3: Resuloğlu; 4: Ahlatlıbel; 5: Polatlı; 6: Troas; 7: Gözlükule; 8: Soli; 9: Tepecik; 10: Kültepe; 11: Alaca Höyük (map created by the author). (TIF) [file pone.0234563.s001.tif]

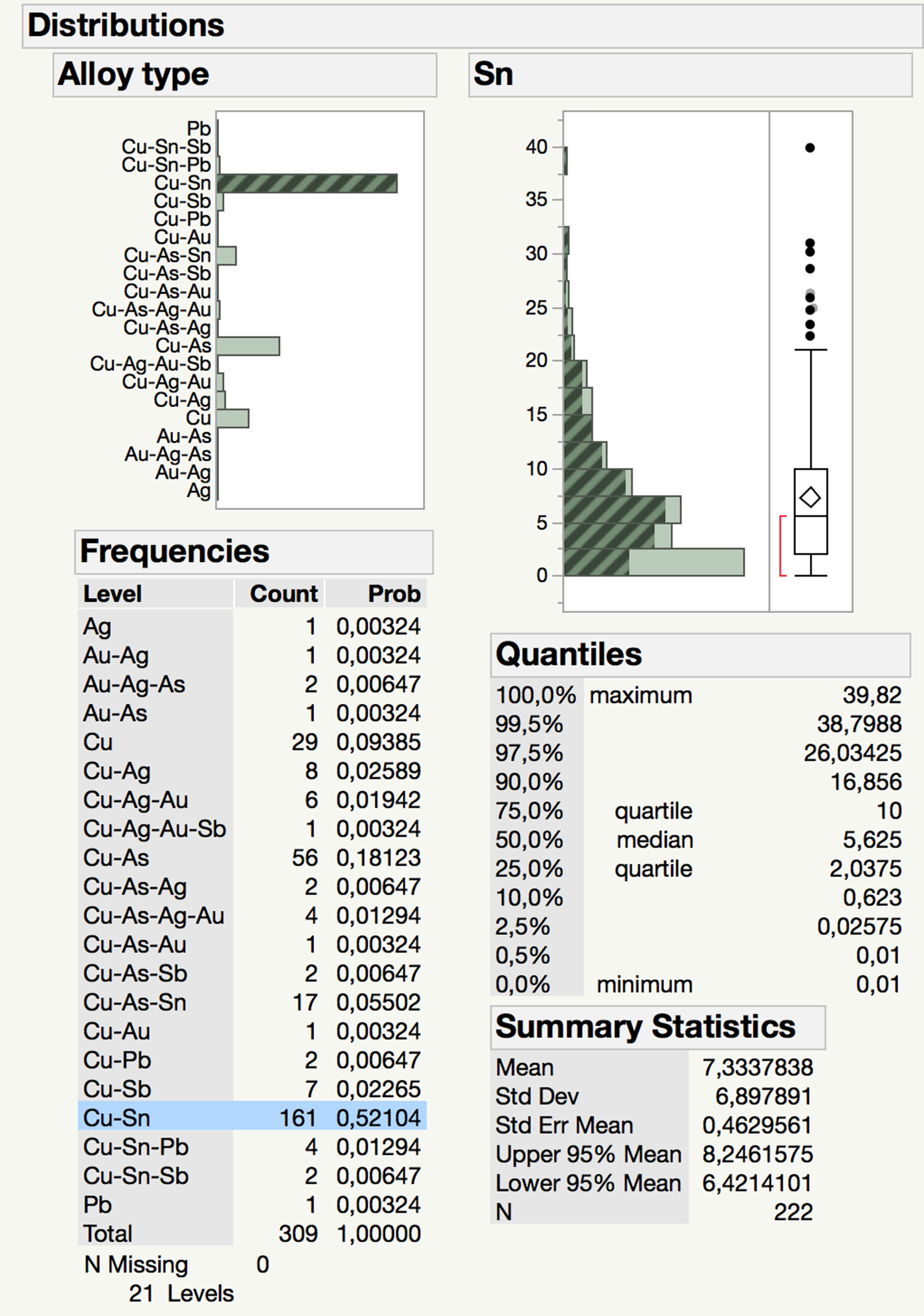

Supplement: S2 Fig — (TIF) [file pone.0234563.s002.tif]

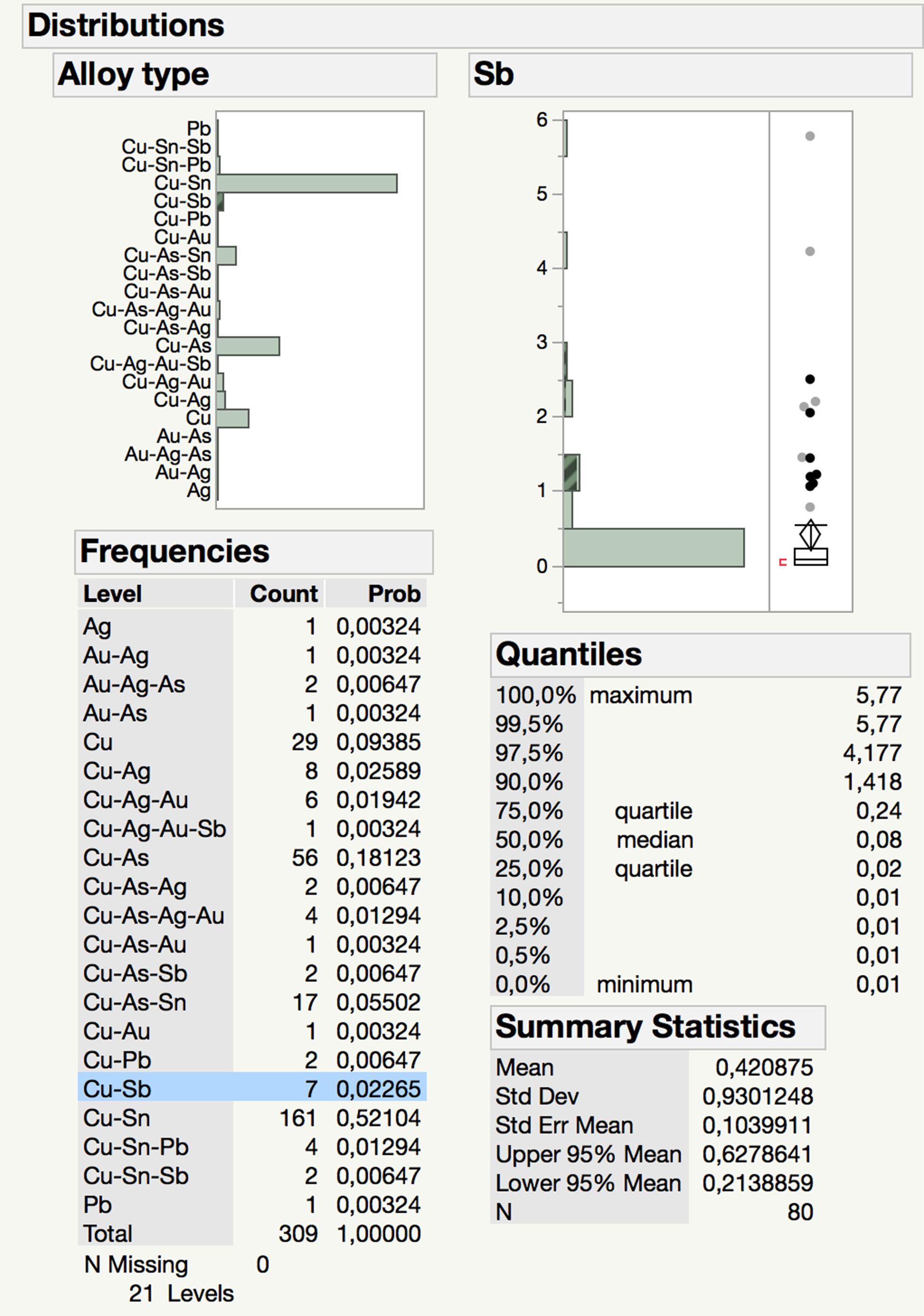

Supplement: S3 Fig — (TIF) [file pone.0234563.s003.tif]

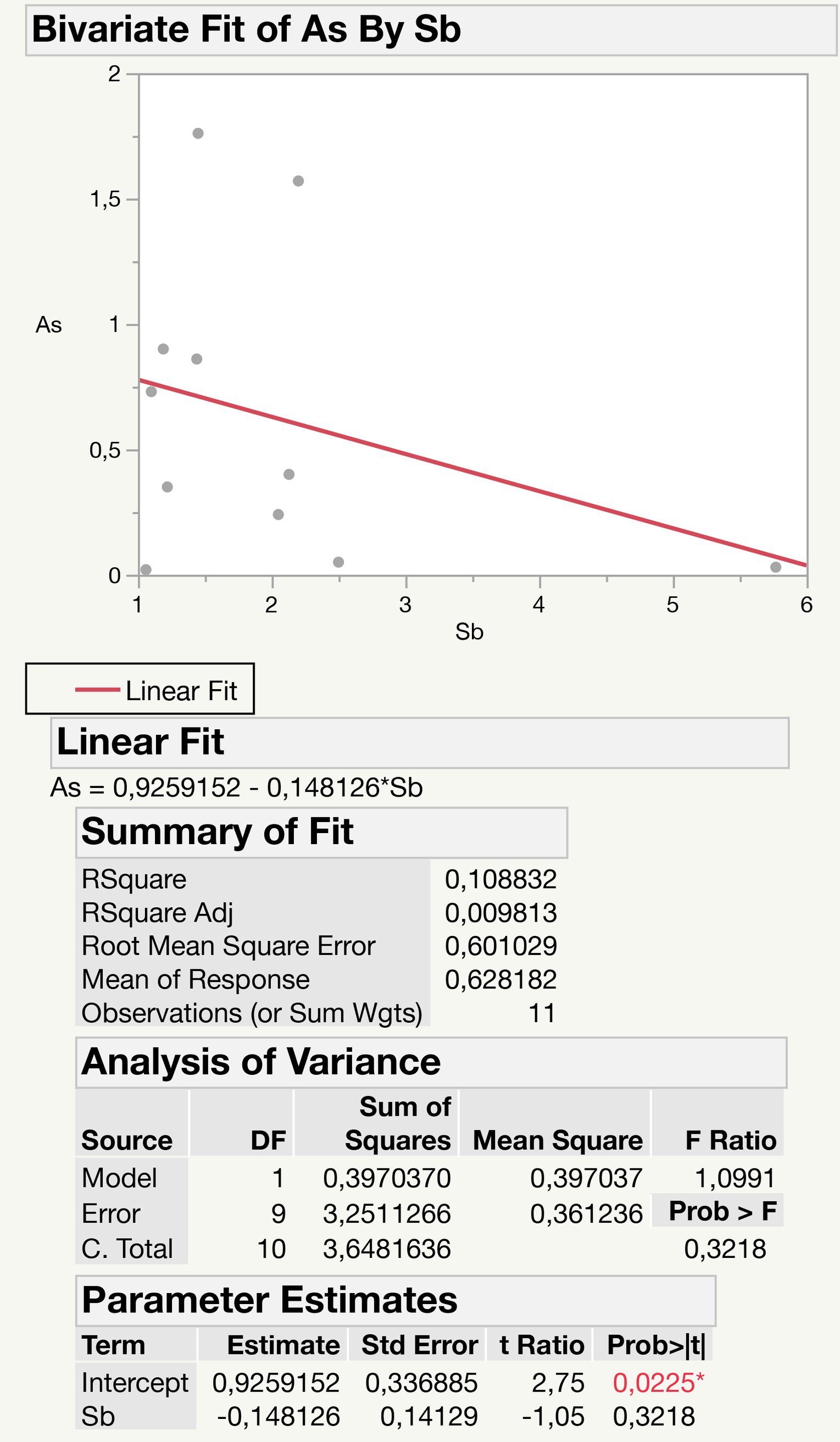

Supplement: S4 Fig — (TIF) [file pone.0234563.s004.tif]

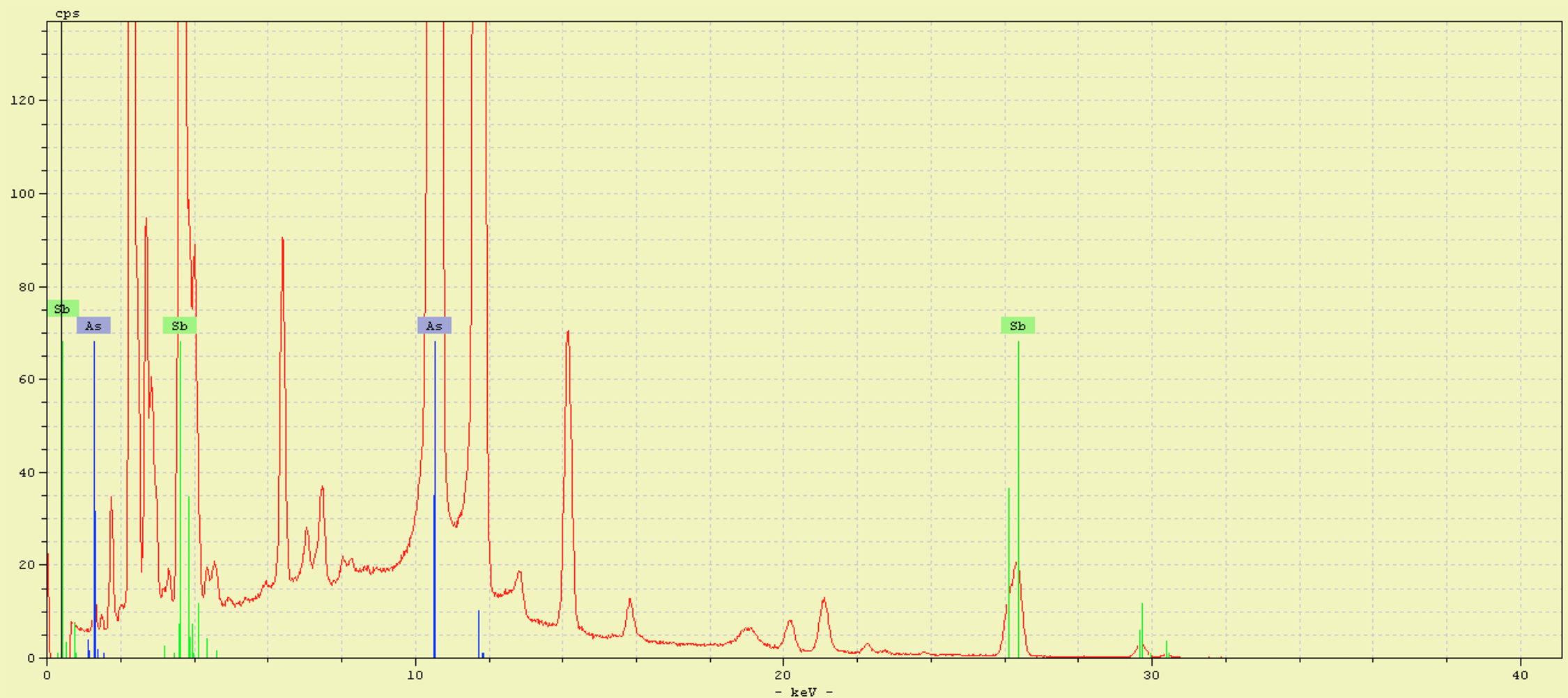

Supplement: S5 Fig — (TIF) [file pone.0234563.s005.tif]
